# Innovative applications of medicinal mushrooms in functional foods and nutraceuticals: a focus on health-boosting beverages

**DOI:** 10.3389/fcimb.2025.1605301

**Published:** 2025-08-20

**Authors:** Jing-ya Yang, Li Tao, Dengji Lou, Nimesha M. Patabendige, Aseni N. Ediriweera, Shujuan Liu, Wenhua Lu, Entaj Tarafder, Sylvie Rapior, Kalani K. Hapuarachchi

**Affiliations:** ^1^ College of Chemistry Biology, and Environment, Yuxi Normal University, Yuxi, Yunnan, China; ^2^ Center for Yunnan Plateau Biological Resources Protection and Utilization, College of Biology and Food Engineering, Qujing Normal University, Qujing, Yunnan, China; ^3^ Department of Plant Pathology, Agriculture College, Guizhou University, Guiyang, Guizhou, China; ^4^ Laboratory of Botany, Phytochemistry and Mycology, Faculty of Pharmacy, Univ Montpellier, Montpellier, France; ^5^ CEFE, Univ Montpellier, CNRS, IRD, EPHE, Natural Substances and Chemical Mediation Team, Montpellier, France; ^6^ College of Biodiversity Conservation, Southwest Forestry University, Kunming, China

**Keywords:** biotechnological applications, clinical trials, health supplements, non-alcoholic beverages, pharmacology, traditional medicine

## Abstract

Mushrooms, which are an integral part of human nutrition and traditional medicine in various cultures, including Asia, the Americas, Africa, and Europe, appear to be an ideal food for a healthy lifestyle. Their rich range of bioactive compounds in certain macrofungi, supported by scientific research and clinical trials, has demonstrated their nutritional and medicinal value. This review covers the historical context, pharmacological efficacy, innovative biotechnological advancements in macrofungal cultivation, and value-added products derived from medicinal mushrooms. It emphasizes the rapidly growing market for mushroom-based beverages, highlighting their role in contemporary health practices and their growing recognition as nutraceuticals and functional foods.

## 1 Introduction

Mushrooms, an incredible group of fungi, have long been celebrated for their nutritional and medicinal properties. With a vast diversity of known edible species and many more within the medicinal category, mushrooms have made significant contributions to human health, culture, and cuisine (Anusiya et al., 2021; Li et al., 2021a; Hamza et al., 2024; Miazzi et al., 2024; Zhou et al., 2024). Traditional medicine has leveraged the therapeutic potential of mushrooms for centuries, with modern research continually validating their benefits (Garcia et al., 2022; Łysakowska et al., 2023; Thomas and Mago, 2024). Mushrooms are rich in bioactive compounds, including polysaccharides, terpenoids, phenolic acids, flavonoids, and alkaloids. These compounds exhibit a spectrum of pharmacological activities, including immunomodulatory effects, anti-inflammatory and antioxidant properties, neuroprotective potential, and anti-hepatoprotective functions (Galappaththi et al., 2022; Kolniak-Ostek et al., 2022; Panda and Luyten, 2022; Zorrilla and Evidente, 2022; Gao et al., 2024; Maaloul et al., 2023; Zhao et al., 2023; Kumar et al., 2024; Tee et al., 2024). Polysaccharides found in fungal cell walls, such as β-glucans, have been shown to modulate immune responses and may help prevent tumor growth, making them important for cancer prevention and treatment. Similarly, compounds like triterpenoids and phenolic acids contribute to liver protection, stress relief, and cognitive health, establishing mushrooms as a promising ingredient for functional beverages that support overall well-being (Rašeta et al., 2020; Zhao and Zheng, 2021; Liu et al., 2024; Setyawan et al., 2024; Zhang et al., 2024a). This growing recognition of mushrooms as a source of health-promoting agents has encouraged innovative ways to integrate them into everyday diets (Niego et al., 2021; Bell et al., 2022; Ogawa et al., 2023; Ashaolu and Adeyeye, 2024).

**Figure 1:**
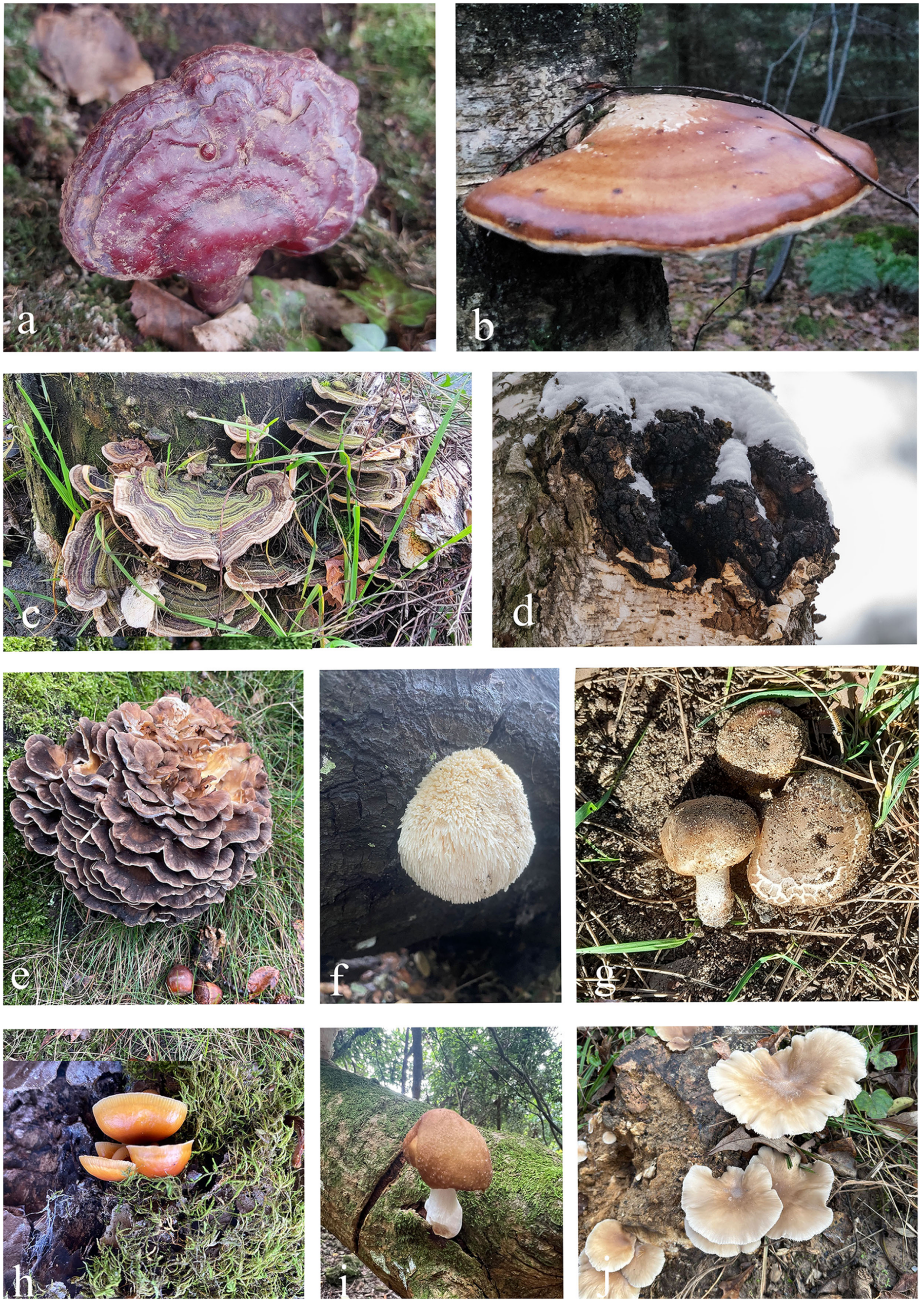
Mushroom species commonly incorporated into functional beverages**. (a–e)** Polyporales **(a)** *Ganoderma lucidum*; **(b)** *Fomitopsis betulina*; **(c)** *Trametes versicolor*; **(d)** *Inonotus obliquus*;**(e)** *Grifola frondosa*). **(f)** Russulales (*Hericium erinaceus*). **(g–j)** Agaricales **(g)** *Agaricus bisporus*; **(h)** *Flammulina velutipes*; **(i**) *Lentinula edodes*; **(j)** *Pleurotus ostreatus* (https://www.inaturalist.org/, the images are used under the license Attribution Non-Commercial-No Derivs 4.0).

Recently, there has been a growing interest in incorporating various mushroom species, not only the well-known medicinal polypores, like *Fomitopsis betulina*, *Ganoderma lucidum*, *Grifola frondosa*, *Inonotus obliquus*, *Polyporus umbellatus*, and *Trametes versicolor*, but also other varieties from the Agaricales, such as *Agaricus bisporus*, *Flammulina velutipes*, and *Lentinula edodes*, as well as *Hericium erinaceus* from the Russulales, into functional beverages (Grienke et al., 2014; Bandara et al., 2015; Hapuarachchi et al., 2021; Fungi Perfecti, 2024; Xu et al., 2024a). Mushroom drinks represent an innovative and growing niche within the broader functional beverage market, appealing to a health-conscious consumer base that seeks natural, holistic alternatives to traditional health supplements (Firenzuoli et al., 2008; Roupas et al., 2012; Hyde et al., 2019; Niego et al., 2023; Grand View Research, 2024). This shift reflects a broader trend toward natural, health-enhancing drinks that provide both unique flavors and therapeutic benefits (Juárez-Aragón et al., 2024). A major challenge in adding mushrooms to beverages is balancing their earthy flavor. To overcome this, natural flavor masking agents and enhancers are used to preserve the health benefits while improving taste. Combining mushrooms with complementary flavors, such as chocolate, vanilla, or spices, helps create appealing taste profiles that enhance the overall sensory experience (Ayseli, 2023). Common mushroom species used in beverages are shown in **Figure 1**.

As interest in functional foods grows, mushrooms are increasingly being utilized in the development of innovative beverages (Contato and Conte-Junior, 2025). Mushroom-based drinks encompass a wide variety of alcoholic and non-alcoholic (NA) options, including mushroom-infused beers, wines, meads, kombuchas, and spirits, as well as NA alternatives such as mushroom teas, coffees, hot chocolates, energy drinks, and wellness tonics, which cater to health-conscious consumers seeking bioactive-rich, plant-based refreshments (Sknepnek et al., 2021; Quan et al., 2022; Sknepnek et al., 2022; Mongkontanawat and Phuangborisut, 2019; Morales, 2024; Sangeeta et al., 2024). These beverages appeal to consumers seeking both unique flavor profiles and the health benefits associated with mushrooms (Haimid et al., 2017; Srivastava et al., 2019). Advancements in extraction and processing technologies are crucial for maximizing the health benefits of mushrooms in beverages. Innovative methods, such as supercritical CO_2_ and ultrasonic-assisted extraction, are developed to enhance the bioavailability and potency of mushroom compounds, ensuring that beverages deliver maximum health benefits (Milovanovic et al., 2021). In addition, microencapsulation is employed to protect sensitive compounds from degradation, resulting in more stable and effective functional ingredients in beverages (Fogarasi et al., 2024).

By combining ancient medicinal knowledge with modern biotechnological advancements, mushroom-based beverages have gained commercial traction, offering therapeutic benefits in convenient and palatable forms. This trend reflects a global shift toward holistic, natural, and sustainable food and beverage choices, driven by consumer demand for products that satisfy taste while promoting health. This review explores the emerging field of mushroom-based beverages, examining the various macrofungal species used in their production, the historical and cultural context of their use, their nutritional and biological properties, and the increasing significance of these beverages in the functional food market. By highlighting the health benefits of mushroom-derived compounds, we explore the potential applications of mushrooms in creating innovative beverages that meet the modern consumer’s desires for taste and well-being.

## 2 Key mushroom species used in beverages, their bioactive compounds, and therapeutic applications

**Figure 2:**
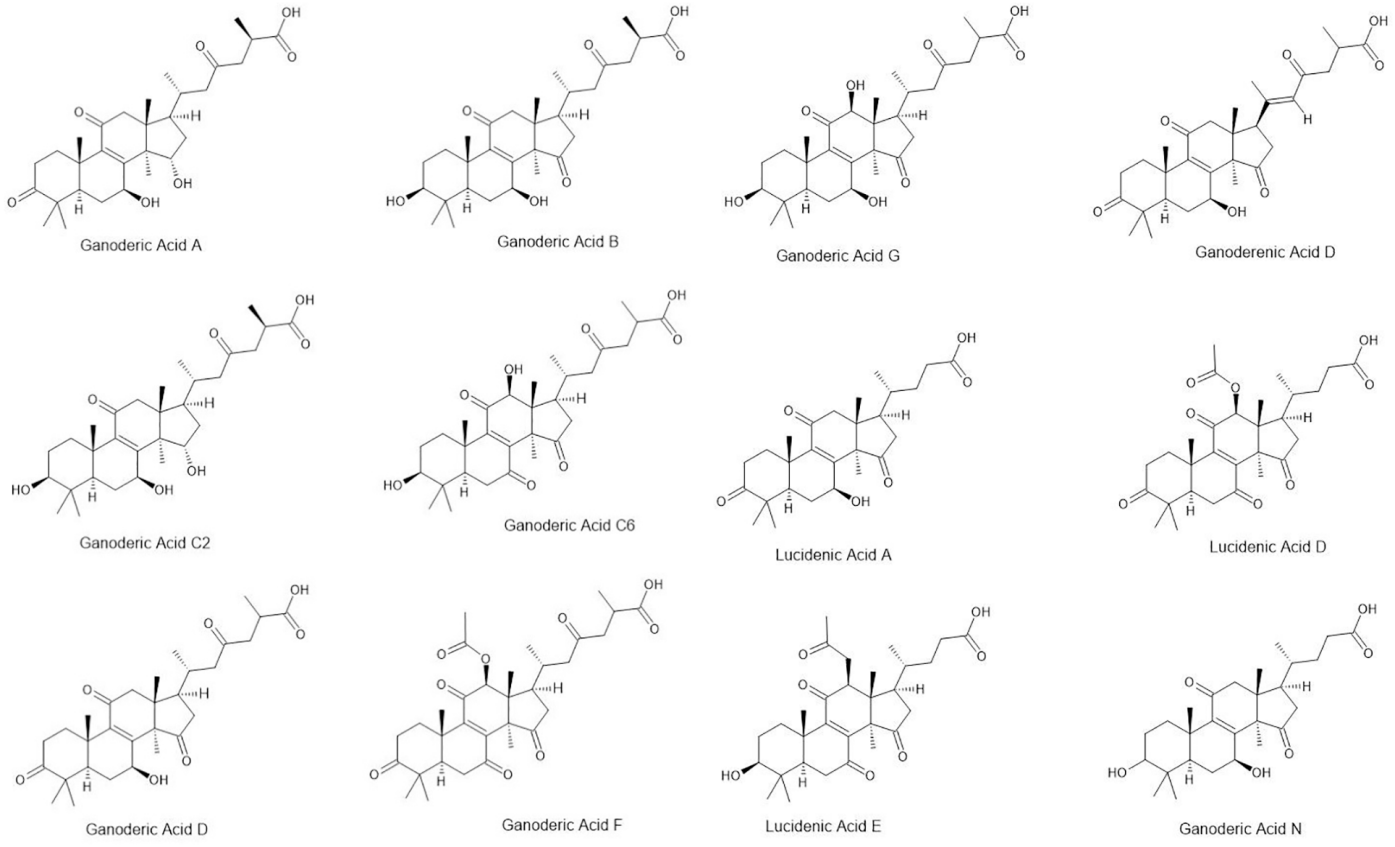
Triterpenoids found in *G. lucidum*–enriched beverages (ACD/ChemSKetch Freeware 12.0, 2012).

*Ganoderma lucidum*, also known as the “Mushroom of Immortality” or Lingzhi, has been a cornerstone of traditional medicine for over two millennia, particularly in Chinese medicine (Jiang et al., 2024; Li et al., 2024a). Valued for its ability to enhance longevity, boost the immune system, reduce stress, and promote overall well-being, *G. lucidum* is commonly used in teas, tinctures, and supplements (Wang et al. 2020a; Swallah et al., 2023; Wu et al., 2024). The therapeutic effects of *G. lucidum* are attributed to its rich content of bioactive compounds, such as polysaccharides (including β-glucans) and triterpenoids (e.g., ganoderic acids) (Galappaththi et al., 2022; Zheng et al., 2023; Zhao et al., 2025; **Figure 2**). Polysaccharides are known for their immune-boosting and anticancer properties, whereas triterpenoids contribute to their anti-inflammatory and antioxidant effects, further supporting their health benefits (Azi et al., 2024; Karunarathna et al., 2024a). Modern research has validated these traditional uses, confirming the potential of *Ganoderma* in immune modulation, reducing inflammation, and protecting the liver. However, its liver-protective benefits are particularly relevant in NA functional beverages, as alcohol consumption can increase the risks of steatosis and hepatitis, counteracting liver health benefits. This makes *Ganoderma* a popular ingredient in NA beverages designed to enhance vitality and well-being (Hu et al., 2020; Desalegn et al., 2024; Wang et al. 2020b). Given the rising global interest in NA functional beverages, *G. lucidum* extracts could be optimized for hepatoprotective formulations targeting populations with metabolic syndrome. However, industry challenges include standardizing bioactive concentrations across commercial products to ensure efficacy.

*Inonotus obliquus*, commonly known as Chaga, is often referred to as the “King of Medicinal Mushrooms” due to its impressive antioxidant properties and therapeutic potential (You et al., 2022b). Traditionally used in teas and as a coffee substitute, *I. obliquus* is highly valued for its immune-boosting effects (Ern et al., 2024; Tee et al., 2024). Melanin, which gives the fungus a dark color, and betulinic acid, known for its anti-inflammatory, liver-protective, and potential anticancer properties, are key bioactive compounds found in the species (Camilleri et al., 2024; Choi et al., 2024; Wang et al., 2024). Recent studies highlight synergistic benefits when combining melanin and betulinic acid with birch-derived compounds, suggesting promising avenues for next-generation nutraceuticals. The mushroom’s detoxification properties align with growing demand for oxidative stress-reducing functional beverages, particularly in sports recovery and urban wellness markets (Lin et al., 2024; Zhang et al., 2024a). Rich in polyphenols, *I. obliquus* offers significant antioxidant and anti-inflammatory benefits, which have contributed to its use in traditional medicine, particularly in Northern Europe and Russia (Wang et al., 2021; Wei et al., 2022; Wagle et al., 2024). Betulinic acid, derived from the birch trees on which *I. obliquus* grows, is associated with antiviral and anticancer effects, further enhancing its therapeutic reputation (Lou et al., 2021; Raal et al., 2024; Wang et al., 2024). *Inonotus obliquus* is widely used in functional beverages due to its combined health benefits, which support immune function and overall vitality (Li et al., 2022; Pawłowicz et al., 2024).

**Figure 3:**
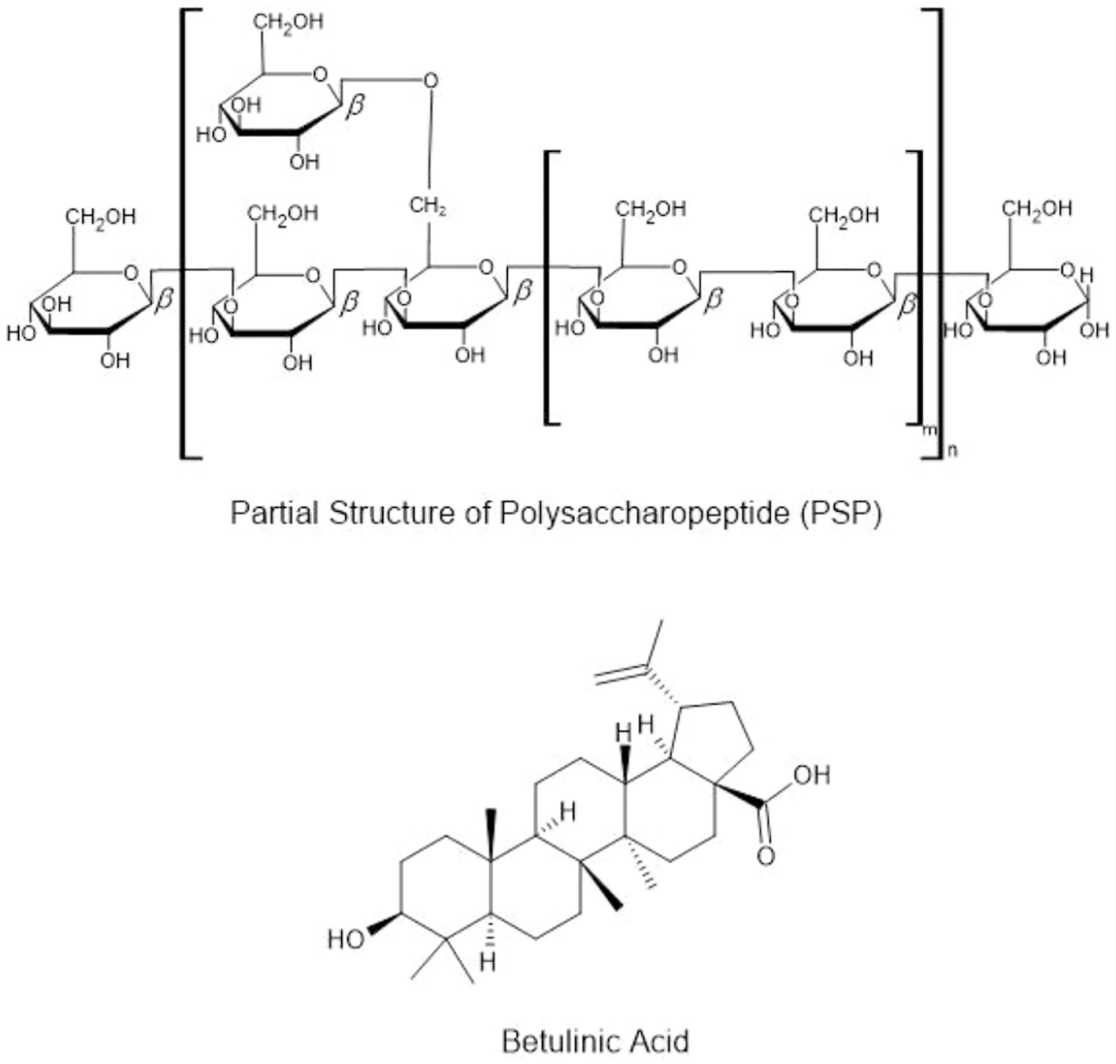
Bioactive compounds from *Trametes versicolor* (PSP) and *Fomitopsis betulina* (betulinic acid).

*Trametes versicolor*, commonly known as Turkey Tail, is a renowned medicinal mushroom widely used in traditional Chinese medicine and modern integrative therapies for its immune-boosting and health-promoting properties (He et al., 2022; Ajibola et al., 2024). These bioactive molecules show particular promise for enhancing gut health, with clinical trials needed to validate PSK’s prebiotic effects in probiotic beverage formulations. While therapeutic benefits are well-established, the industry faces challenges in developing cost-effective, scalable methods for PSK extraction that could enable mass production of *T. versicolor*–enriched functional drinks (Muto and Yamaguchi, 2014; Lo et al., 2020; Lowenthal et al., 2023; Tang et al., 2023; Darshan et al., 2024; **Figure 3**). In addition, its potential benefits for gut health have increased its popularity in teas, dietary supplements, and wellness beverages designed to promote overall health (Zhang et al., 2015; Janjušević et al., 2018; Cerig, 2021).

**Figure 4:**
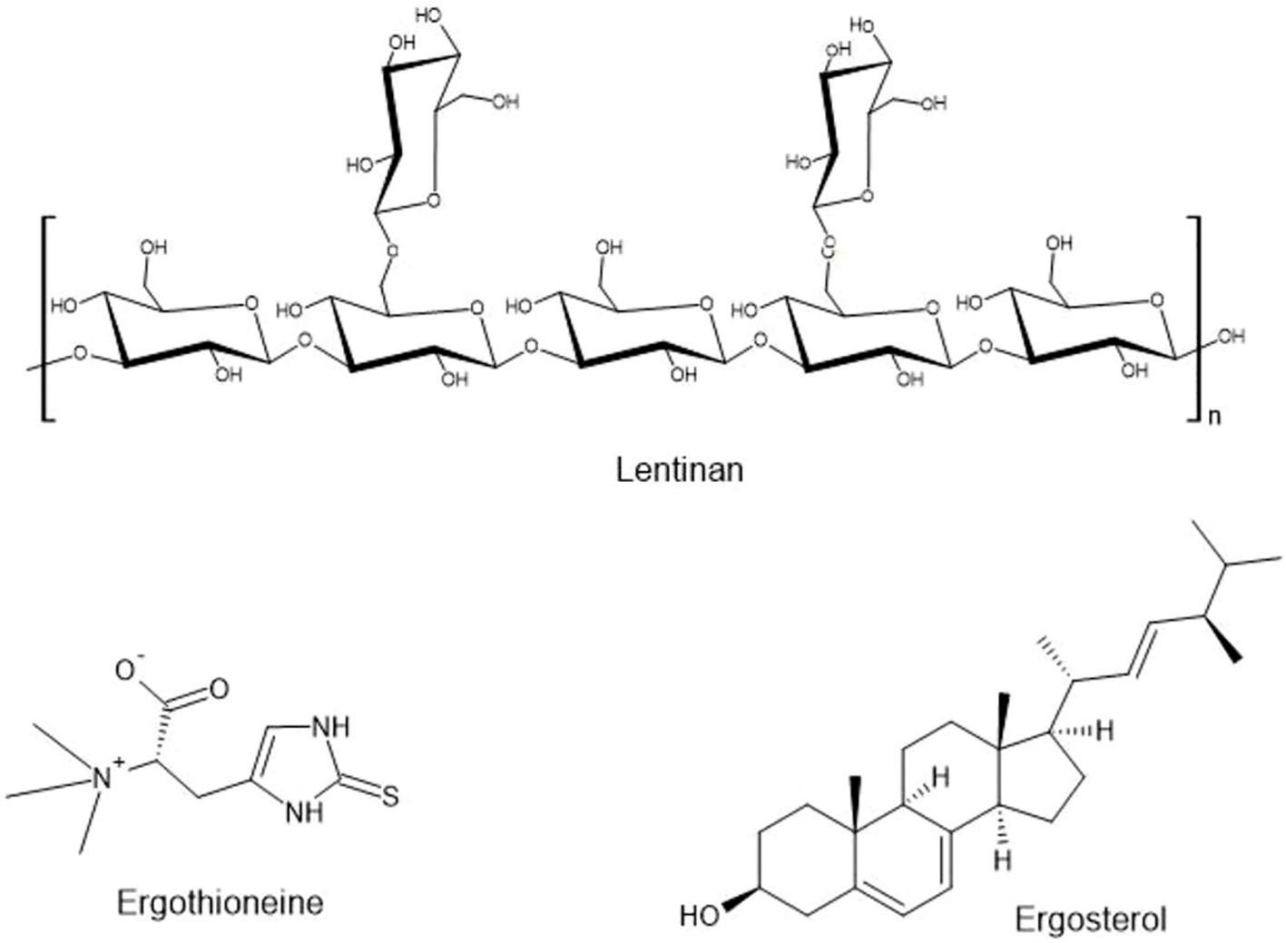
Bioactive compounds from *Lentinula edodes* (lentinan), *Pleurotus* sp. (ergothioneine), and *Agaricus* sp. (ergosterol).

*Lentinula edodes* (Shiitake) is widely used in culinary applications, particularly in soups and teas, but it also offers significant health benefits (Diallo et al., 2020; Timm et al., 2022; Ahmad et al., 2023). Lentinans, polysaccharides produced by *L. edodes* reported to possess immune-boosting and cholesterol-lowering effects (Sheng et al., 2021; Wang et al., 2023; Sahib et al., 2024; Xu et al., 2024a; **Figure 4**). Regular consumption of *L. edodes* has been linked to improved cardiac health, enhanced immunity, and reduced inflammation, making it a popular choice in functional beverages (Dai et al., 2015; Roszczyk et al., 2022; Zhang et al., 2022). However, current research lacks clear dose-response data for these compounds in beverage applications, representing a critical knowledge gap. Innovative product development could explore fortifying plant-based milk alternatives with *L. edodes* extracts, creating hybrid products that combine the nutritional benefits of plant-based milk with growing consumer demand for dairy-free options.

**Figure 5:**
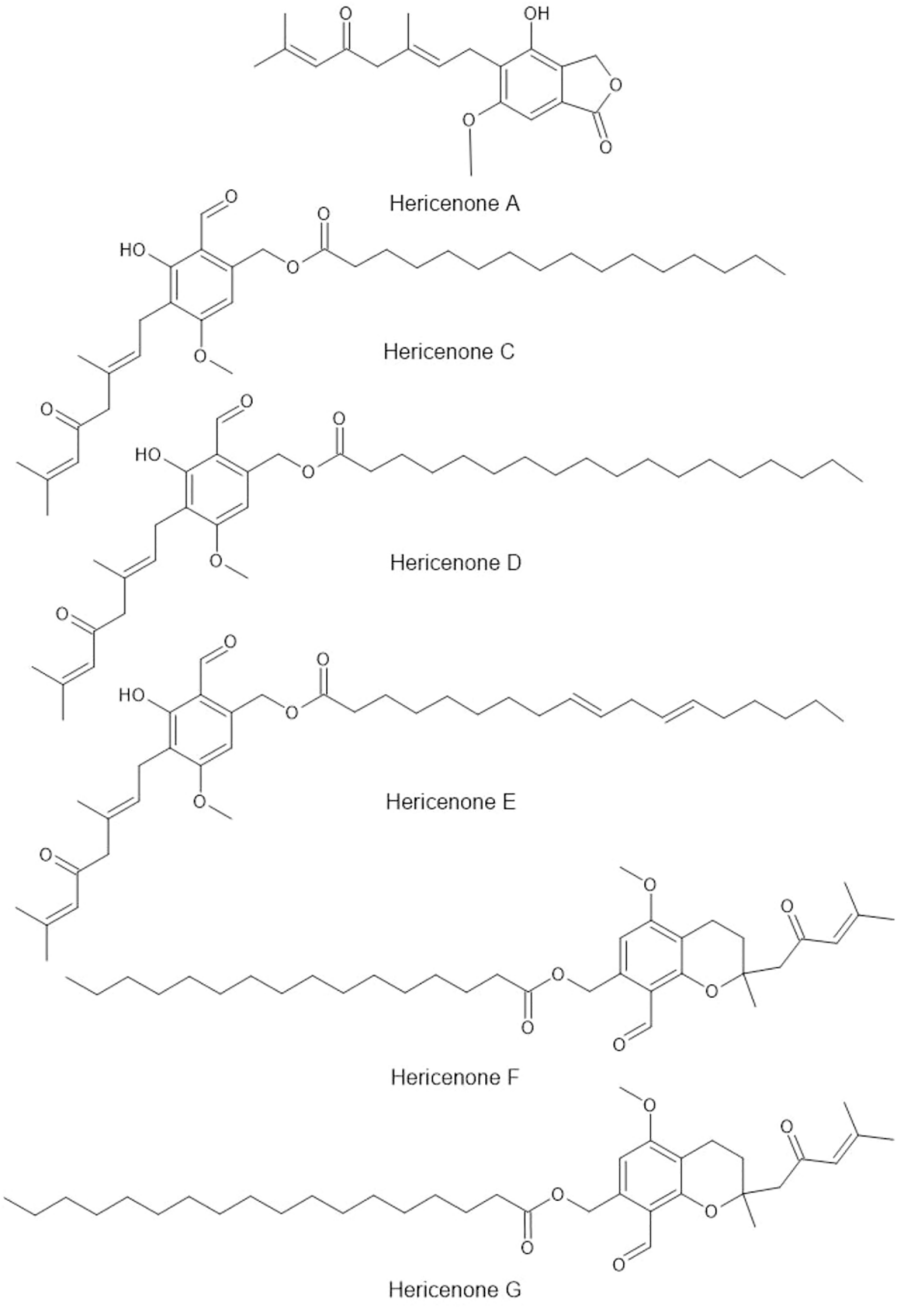
Bioactive compounds from *Hericium erinaceus*.

*Hericium erinaceus* (Lion’s Mane) is a unique mushroom known for its potential cognitive benefits (Saitsu et al., 2019; Cortonesi et al., 2023; Spano et al., 2024). Often consumed as a tea or in supplement form, *H. erinaceus* is prized for its ability to support brain health and promote nerve regeneration (Lin et al., 2023; Opanuga and Hossain, 2024). The mushroom contains hericenones and erinacines, compounds that stimulate the production of nerve growth factor, which is essential for the maintenance and growth of neurons (Kostanda et al., 2024; Qiu et al., 2024; Jianzhao et al., 2024; Johansen et al., 2019; **Figure 5**). The cognitive-enhancing properties of this fungus position it ideally for development in brain health beverages targeting aging populations and consumers seeking nootropics. As a result, *H. erinaceus* has gained popularity among individuals seeking to enhance memory, focus, and overall cognitive function (Roda et al., 2023; Bizjak et al., 2024). However, formulation challenges persist regarding the stability of hericenones in liquid products, requiring additional research to ensure consistent potency in ready-to-drink formats.

Polysaccharides are complex carbohydrates found in many medicinal mushrooms, including *G. lucidum*, *I. obliquus*, and *T. versicolor* (Gao et al., 2024; Wang et al., 2024; Wold et al., 2024). These compounds are highly valued for their immune-boosting, anti-inflammatory, and anticancer properties (Yu et al., 2023; Flores et al., 2024). Polysaccharides like β-glucans have been shown to stimulate immune cells, enhance resistance to infections, and reduce inflammation (Murphy et al., 2022; Vetter, 2023; Mizuno and Minato, 2024). They play a central role in the therapeutic effects of mushrooms and are a key reason these mushrooms are incorporated into health-promoting beverages. Nevertheless, the immune-modulating β-glucans from medicinal mushrooms face challenges related to bioavailability in beverages. Encapsulation technologies like nanoemulsions or liposomal delivery systems could significantly enhance their absorption while maintaining stability in liquid formulations. Nanoencapsulation trials with lentinan (from *L. edodes*) demonstrate 2.3 times higher gut absorption in murine models (Zhao et al., 2024)—a strategy transferable to human beverage formulations.

Triterpenes are a group of bioactive compounds found in *G. lucidum* and *I. obliquus*, known for their potent anti-inflammatory, antioxidant, and liver-protective effects (De Oliveira et al., 2024; Han et al., 2024; Zhao et al., 2025). Triterpenes are thought to help protect the liver from toxins, reduce inflammation in the body, and support overall health. These compounds also contribute to the unique bitter flavor of certain mushrooms, making them ideal for blending into medicinal teas and beverages (Chen et al., 2021; Zhao et al., 2023; Raza et al., 2024; Zhao et al., 2023a; Zhao et al., 2023b). While triterpenes (e.g., ganoderic acids) offer potent anti-inflammatory benefits, their characteristic bitterness poses formulation hurdles for ready-to-drink (RTD) products—strategic use of natural flavor maskers (e.g., monk fruit and stevia) or microencapsulation may improve palatability.

Phenolic compounds, which are potent antioxidants, are found in *L. edodes* and *T. versicolor* (Tel-Çayan et al., 2021; Morales et al., 2024). These compounds help reduce oxidative stress, a key factor in aging and various chronic diseases. By scavenging free radicals and reducing inflammation, phenolic compounds support cardiovascular health, cognitive function, and overall well-being (Krsmanović et al., 2023; Darshan et al., 2024). As a result, mushrooms rich in phenolic compounds are frequently used in functional beverages targeting antioxidant support. As mushroom-derived phenolics demonstrate strong antioxidant capacity, synergistic combinations with berry extracts (e.g., blueberries and aronia) could create dual-action functional beverages with enhanced Oxygen radical absorbance capacity (ORAC) values and improved flavor profiles. Hybrid mushroom-berry beverages could leverage the FDA’s “qualified health claim” pathway for antioxidant effects, accelerating US market entry.

β-Glucans have been extensively studied for their ability to modulate the immune system, enhance the body’s ability to fight infections, and inhibit tumor cell growth (Timm et al., 2023; Bu et al., 2024; Qu et al., 2024). β-Glucans are a primary reason why mushrooms like *G. lucidum* and *I. obliquus* are considered key ingredients in health-conscious beverages. While terpenes contribute to some of the therapeutic properties of mushrooms, other classes of volatile compounds, such as those identified in *Marasmius alliaceus* and *Tuber* spp., as well as in other fungi, play a key role in their distinctive aroma and flavor profiles. These compounds, including (Z)- and (E)-1,3-octadiene, enhance the sensory experience and are often explored for their potential applications in creating beverages with therapeutic effects (Breheret et al., 1997, Breheret et al., 1998; Rapior et al., 1997, Rapior et al., 2000; You et al., 2022a; Yeong et al., 2024). Proteins and peptides are another class of bioactive compounds present in all the mentioned mushrooms. These compounds contribute to the nutritional profile of medicinal mushrooms, providing a source of protein, essential amino acids, and other nutrients that support overall health. Peptides also play a role in immune modulation, tissue repair, and anti-aging, making them valuable in beverages designed for overall health enhancement (Ayimbila and Keawsompong, 2023; Ahmed et al., 2024; Drzewiecka et al., 2024; Li et al., 2021, Li et al., 2024b, c). Mushroom proteins provide complete amino acid profiles. As demand for plant-based alternatives grows, mushroom-derived peptides are poised to compete with dairy/whey proteins in sports nutrition and wellness beverages, offering sustainability advantages.

## 3 Mushroom-based non-alcoholic beverages, research, and innovations

The resurgence of interest in medicinal mushrooms has led to the development of a wide array of mushroom-infused drinks, ranging from teas and coffees to more complex formulations such as smoothies and energy drinks. For example, mushroom coffee blends have gained significant popularity due to their ability to combine the energy-boosting effects of caffeine with the adaptogenic properties of mushrooms (Roupas et al., 2012). The production of mushroom drinks involves sophisticated extraction and formulation techniques to ensure the bioavailability and stability of the active compounds, which can be sensitive to heat and other environmental factors. Advances in biotechnology have enabled the development of extraction methods that maximize the retention of these bioactive compounds, ensuring that the final products deliver the intended health benefits (Firenzuoli et al., 2008). For instance, advanced dual-extraction techniques now integrate novel approaches, such as ultrasonic-assisted extraction and microwave-assisted extraction, with traditional methods using alcohol and water. These methods enhance the recovery of bioactive compounds, including water-soluble polysaccharides and alcohol-soluble triterpenes, from medicinal mushrooms such as *G. lucidum* and *I. obliquus* (Zjawiony, 2004; Leong et al., 2021; Wang et al., 2022; Deveci et al., 2024). Furthermore, fermentation is gaining popularity in the functional beverage industry to enhance the nutritional value and flavor of mushroom drinks. These fermented beverages, often enriched with probiotics, offer additional health benefits. Mushroom-infused kombucha and kefir combine the advantages of fermentation with the health properties of mushrooms, promoting digestive and immune health (Kruk et al., 2021). As the demand for functional beverages continues to grow, the exploration of mushroom-based NA drinks has become an exciting frontier. In the following section, we will discuss the latest research and innovations in this field, highlighting various mushroom-infused beverages that have a significant impact on the market. From mushroom teas to smoothies, these drinks are poised to offer unique health benefits while providing consumers with an alternative to traditional beverages.

### 3.1 Coffee

Mushroom coffee represents a fusion of traditional coffee with the therapeutic properties of medicinal mushrooms. Typically, this involves blending coffee with mushroom extracts in a 1:1 ratio, such as *G. lucidum*, *H. erinaceus*, and *I. obliquus*, which are known for their cognitive and immune-enhancing effects (Hobbs, 2017; Smith, 2021). The process typically involves extracting bioactive compounds from mushrooms, which are then combined with ground coffee beans or instant coffee, resulting in a beverage that retains the familiar taste of coffee while offering additional health benefits (Wani et al., 2010; Kała et al., 2024). Mushroom coffee is gaining traction due to its potential to reduce the adverse effects of caffeine, such as jitters and crashes, while providing sustained energy and mental clarity (Roupas et al., 2012; Blundell and Camilleri, 2025). The adaptogenic properties of mushrooms contribute to stress reduction and improved overall well-being (De Silva et al., 2012).

Market trends indicate a growing demand for mushroom-infused coffees, driven by consumers seeking functional beverages that offer more than just a caffeine boost. This trend aligns with the broader shift toward health and wellness products, with mushroom coffee emerging as a key player in the functional food and beverage sector (Wasser, 2014). The mushroom coffee market, valued at approximately $2.5 billion in 2022, is projected to grow at a compound annual growth rate (CAGR) of 6.8% over the next decade, reflecting its increasing popularity and significant contribution to the functional beverage industry (Myshroomz, 2023). Neurohealth-focused formulations (e.g., for menopause-related brain fog) could capture 20-30% of the functional coffee market by 2030, pending clinical validation of mushroom-caffeine synergies.

*Agaricus blazei* (royal sun agaric mushroom) hot water extract is incorporated into coffee and chocolate drinks for its health benefits, including support for digestive health and immune function (Frost, 2016). An instant coffee produced from *A. subrufescens* (almond mushroom) and its extract, when mixed with hot water, is also used as a beverage and a food supplement (Gyorfi et al., 2010). Cordyceps coffee, developed with extracts from *Cordyceps*, *Phellinus*, and *Inonotus* mushrooms, was evaluated for its biological and quality properties. It showed higher polyphenol content and antioxidant activity compared to raw *Cordyceps* mushrooms. Although it had lower β-glucan and Cordycepin levels than the raw mushrooms, it maintained the coffee aroma without off-flavors. The study suggests that *Cordyceps* coffee is a functional beverage containing beneficial compounds, such as cordycepin and β-glucan (Song, 2020). A patent describes a method for producing a coffee beverage containing *G. lucidum*. The method involves preparing a *G. lucidum* extract and mixing it with scorched rice extract and coffee flavor extract. The resulting beverage is nutrient-rich, has improved flavor and aroma, and reduces caffeine intake, offering a healthier coffee alternative (Korea Patent KR2024062784A, 2024). Furthermore, flavor-masking technologies using mushroom-rice blends may enable 50% faster regulatory approval for novel beverages in strict markets like the EU and Japan.

The Fungi Perfecti Company (USA) created MUD/WTR, a coffee alternative designed to boost vitality, by blending the mycelium of *I. obliquus*, *Cordyceps militaris*, *H. erinaceus*, and *G. lucidum* with other components (Yan et al., 2023). Green coffee beans (Indonesian Mandheling) were fermented with mushrooms (*Phellinus linteus*, *H. erinaceum*, and *G. lucidum*) or molds (*Monascus purpureus*, and *Monascus ruber*) using solid-state culture to enhance their functional properties. Roasted coffees from mold-fermented beans exhibited higher yields of polyphenols and flavonoids, enhanced antioxidant activity, and increased chlorogenic acid in hot-water extracts. Notably, *M. ruber* showed the strongest macrophage-stimulating and mitogenic activities. These extracts inhibited nitric oxide production without cytotoxicity. Caffeine levels remained unchanged, indicating that mold-fermented coffees could serve as functional beverages with industrial potential (Shin et al., 2013). Beyond mushroom coffee, other fungal-based coffee beverages utilize yeasts such as *Saccharomyces cerevisiae* and *Torulaspora delbrueckii* in fermentation processes to enhance flavor complexity, improve shelf stability, and introduce potential health benefits. Fermented coffee, for example, leverages these yeasts to develop unique sensory profiles while retaining the natural richness of coffee (Martins et al., 2022).

### 3.2 Teas

Mushroom teas have a rich history in traditional medicine, particularly in Asia, where they have been consumed for centuries as therapeutic beverages. Traditional formulations often involve the use of dried or powdered mushrooms, such as *G. lucidum*, *T. versicolor*, and *I. obliquus*, brewed with hot water to extract their bioactive compounds. These mushrooms are revered for their potential to enhance immunity, reduce inflammation, and combat fatigue (Zhao et al., 2020). Standardized hot-water extraction protocols for *G. lucidum* teas could increase bioactive yield by 30%–40% while reducing production time, based on recent ultrasonic-assisted extraction trials (Zhang et al., 2024b). In modern formulations, mushroom teas have evolved to include blends with various herbs, spices, and even other teas, such as green or black tea, catering to diverse consumer preferences while enhancing overall health benefits (Wachtel-Galor and Benzie, 2011). Furthermore, blending *T. versicolor* with matcha may create dual-functional teas that target both gut health (via PSK) and metabolic benefits (from EGCG), capitalizing on the $12 billion global tea wellness market.

The benefits of mushroom teas are primarily derived from their rich content of polysaccharides, triterpenoids, and antioxidants, which contribute to their immunomodulatory, anticancer, and anti-inflammatory properties (Wasser, 2014). Namibian *Ganoderma*, although largely untapped in Africa, shows promise for medicinal tea applications. A study identified and cultivated local species, evaluating their water absorption, solubility, and phenolic content. Infusions made from cultivated *Ganoderma* had higher total phenolics, condensed tannins, and antioxidant activity than those from wild counterparts, suggesting enhanced nutraceutical potential. Both wild and cultivated *G. lucidum* exhibited potent antioxidant activity, supporting their potential use in nutraceutical tea formulations (Hamwenye et al., 2022). African-grown *G. lucidum* exhibits 18% higher antioxidant activity than its Asian variants in laboratory studies—a potential Unique Selling Proposition (USP) for “single-origin” medicinal tea branding in premium markets. Consumers are increasingly drawn to these teas for their potential health benefits, particularly in boosting immunity and improving mental clarity. The rising popularity of functional beverages has positioned mushroom teas as a preferred choice among health-conscious individuals, particularly those looking for natural, caffeine-free alternatives to traditional teas (Chang and Miles, 2004). Developing caffeine-free adaptogenic tea blends (e.g., *G. lucidum* + *M. chamomilla*) could capture the growing $7.8B stress-relief beverage sector, particularly among Gen Z consumers. The adaptability of mushroom teas to various flavor profiles, combined with the growing awareness of their health benefits, has contributed to their widespread acceptance in both traditional and modern markets (**Table 1**).

**Table 1:** Health benefits, methods, and applications of mushroom-based teas.

Mushroom species	Study	Health benefits	Methods	Findings	Reference
*Agaricus blazei* Murrill, green tea	Solid green tea beverage optimization	Immune support, flavor improvement	Beverage optimization parameters	Optimized formula achieved good solubility, flavor, and appearance, claiming benefits for immune support and digestive health	Cheng et al. (2019)
*Auricularia polytricha* (black jelly mushroom)	Functional kombucha drink	Cardiovascular support, weight loss	Kombucha fermentation with black jelly mushroom	Combined benefits of kombucha and black jelly mushroom offer cardiovascular support and weight loss, with commercialization potential	Rahman and Muhammad (2022)
*Flammulina velutipes*	Flavor enhancement in green tea	Flavor enhancement	Green tea fermentation with *F. velutipes*	Fermentation reduced green notes and enhanced nutty, chocolate-like aromas with the emergence of alkylpyrazines	Rigling et al. (2021)
*Fomes fomentarius*	Traditional use of *F. fomentarius* tea	Bladder disorder treatment, anticancer	Tea preparation	Traditionally used as a tea in Chinese medicine for treating bladder disorders and certain cancers.	Papp et al. (2017); Campi et al. (2024)
*Fomitopsis betulina*	Betulinus tea and extract	General health benefits	Tea, tonic, tincture preparation	Betulinus extract is traditionally prepared as tea or tonic for general well-being.	Pleszczyńska et al. (2017)
*Ganoderma lucidum*	Synergistic effect of green tea and *Ganoderma lucidum*	Anticancer (breast cancer), anti-inflammatory	*In vitro* cell studies, growth inhibition assays	GTE and GLE inhibited breast cancer cell growth, adhesion, migration, and invasion by downregulating c-myc expression and suppressing uPA secretion.	Thyagarajan et al. (2007)
*G. lucidum*, *Cymbopogon citratus*, *Lippia multiflora*	Composite tea formulation	Antioxidant, sensory appeal	Tea formulation and sensory testing	Formulation A (50% *G. lucidum*) preferred for its antioxidant properties, higher protein, crude fiber, and ash content, compared to Lipton tea	Dzah (2015)
Various *Ganoderma* sp.	Traditional mushroom tea	Immunity enhancement, anti-inflammatory	Brewing dried/powdered mushrooms with hot water	Traditional use of *G. lucidum*, T*. versicolor*, and *I. obliquus* for enhancing immunity, reducing inflammation and combating fatigue	Zhao et al. (2020)
*G. lucidum*, *Monascus purpureus*	Functional black tea development	Nutritional enhancement, GABA content	Mixed fermentation	Functional black tea enriched with GABA and exopolysaccharides, improving its nutritional properties and health advantages	Gao (2013)
*G. lucidum*	Sensory optimization of tea beverage	Antioxidant, flavor preservation	Enzymolysis, GC-MS analysis	Optimal formula preserved aroma (2-ethylhexan-1-ol) and achieved 120-day shelf life with good sensory quality	Liu et al. (2017)
*G. resinaceum*	*G. resinaceum* tea antioxidant Properties	Antioxidant, anticancer	Antioxidant and cell growth inhibition assays	Demonstrated significant antioxidant activity and dose-dependent inhibition of breast cancer cell growth	Ghosh et al. (2023)
*Ganoderma*-infused white tea	Advanced antioxidant capacity assessment	Antioxidant	Electrochemical methods, DPPH, ABTS, FRAP	CB-AuNP-modified electrodes provided rapid and reliable antioxidant capacity measurements, linking results to polysaccharide content.	Zhao et al. (2024)
*Inonotus obliquus*	Chaga tea benefits	Immune support, antioxidant, anticancer	Tea and tincture preparation	Chaga tea demonstrated immune-boosting properties, antioxidant activity, and anticancer effects	Balandaykin and Zmitrovich (2015)
*Phellinus igniarius*	Optimization of *P. igniarius* tea production	Antioxidant	Submerged fermentation optimization	Optimized conditions increased polysaccharide content (0.064 g/100 mL) and antioxidant activity.	Shao et al. (2016)
*Grifola frondosa*	Tea for digestive and spleen health	Digestive health, spleen ailments, calm nerves	Tea preparation	Traditionally used to treat spleen and stomach issues, calm nerves, and improve overall digestive health.	Hobbs (1995)
*Phellinus igniarius*, *Eurotium cristatum*	Functional mulberry black tea	Antioxidant, flavonoid enhancement	Fermentation with *P. igniarius* and *E. cristatum*	*P. igniarius* tea had higher polysaccharide content, whereas *E. cristatum* tea exhibited increased flavonoid levels, enhancing functional properties and aroma.	Ma et al. (2022)
*Polyporus umbellatus*	Instant tea powder	Respiratory health, anemia prevention	Tea powder formulation	Mushroom-based tea granules claimed to nourish blood and prevent anemia while offering potential benefits against respiratory tract infections	Han (2013); Liu (2014)
*Pleurotus sajor-caju*	Fermentation of green tea with *P. sajor-caju*	Flavor enhancement, antioxidant	Green tea fermentation	Fermentation reduced bitterness and enhanced fruity aromas while improving ABTS-free radical scavenging ability.	Shi et al. (2009)
	Green tea fermentation with *P. sajor-caju*	Flavor improvement	Fermentation process analysis	Reduced polyphenol content, improving flavor by reducing bitterness while enhancing floral and fruity aromas (linalool and geraniol).	Su et al. (2022)
*Phylloporia ribis*	Development of GABA Radix Arctii fermented hypha tea	Supports health; convenient for modern lifestyles	Modern fermentation; absorption of GABA-enriched brown rice during growth	Produces a tea with light sweetness, mellow flavor, and ease of preparation for daily use	Xing, 2023
*Trametes versicolor*	Turkey tail mushroom tea	Anti-inflammatory	Tea preparation	Traditionally used for reducing inflammation throughout the body	Hobbs (2004)

An *Agaricus blazei* Murrill–flavored green tea solid beverage was developed with optimized preparation parameters, including a 0.20-mm powder size, 1:20 material-to-water ratio, 90°C extraction for 35 min, and freeze-drying at −35°C. The optimal formula included 15 g of mixed powder (green tea: *A. blazei* Murrill 2:1), 1.0 g of sugar, 0.08 g of citric acid, and 0.03 g of β-cyclodextrin, resulting in a product with good solubility, bright color, and pleasant flavor (Cheng et al., 2019). The optimized 0.20-mm powder size and freeze-drying protocol (−35°C) can be adapted for other medicinal mushrooms, potentially reducing production costs by 15%–20% for small-scale manufacturers. Kombucha tea, fermented with SCOBY, offers health benefits such as antidiabetic, anti-inflammatory, and antioxidant effects. *Auricularia polytricha* (black jelly mushroom) offers additional health benefits, including cardiovascular support and potential weight loss benefits. Combining both creates an innovative, functional drink with potential for commercialization, although it is not yet available on the market (Rahman and Muhammad, 2022). Dual-fermented *Auricularia*-SCOBY beverages may address the 43% growth in consumer demand for gut-health products in Southeast Asian markets (Ganchoudhuri, 2021).

Suitable parameters for developing an instant tea product from *C. militaris* extract using convection drying were determined to be 40% maltodextrin, 6% extract, a drying time of 15 h, and a temperature of 70°C. Under these conditions, the cordycepin content was 97.274 µg/g of dry weight, and sensory evaluation resulted in a score of 16.4 out of 20 points. These findings highlight the effectiveness of using maltodextrin in convection drying to produce a uniform powder suitable for beverage applications (Nhan et al., 2022). The cordycepin content at 97.274 µg/g establishes a new standard for bioactive retention in instant mushroom teas, comparable to that of fresh brews (p < 0.05). A patent by Lovely Professional University, India, described a functional mushroom tea made from *C. militaris* blended with turmeric, green tea, and black pepper. The tea was designed to enhance immune function, provide anticancer properties, and improve the bioavailability of active ingredients. It also supported sustainable agriculture and local farming (Indian Patent, IN202411077328A, 2024). Combining regional mushrooms (e.g., Indian *C. militaris*) with Ayurvedic ingredients creates Intellectual Property (IP)-protected formulations with two to three times higher local market penetration potential. Western consumers prefer tea with minimal green flavor, and edible basidiomycetes, such as *Flammulina velutipes* (enokitake), have been used to enhance the flavor of tea naturally. After fermenting green tea with enokitake, the green and floral notes were replaced by nutty and chocolate-like aromas, with key compounds like 2-ethyl-3,5-dimethylpyrazine emerging. This compound, not previously reported in basidiomycetes, is likely produced from vitamin B1 and sodium acetate, along with other alkylpyrazines (Rigling et al., 2021). Pyrazine development during basidiomycete fermentation offers a natural pathway to create chocolate-flavored functional teas without additives.

*Fomes fomentarius* is used in traditional Chinese medicine or as tea to treat bladder disorders and cancers (Papp et al., 2017; Campi et al., 2024). *Piptoporus betulinus* (= *Fomitopsis betulina*) extract is used to make tea, tonic, or tincture (Pleszczyńska et al., 2017).

Green tea extract (GTE) and *G. lucidum* extract (GLE) synergistically inhibit the growth, adhesion, migration, and invasion of breast cancer cells. This combined effect is mediated by downregulating c-myc expression and suppressing urokinase plasminogen activator (uPA) secretion, highlighting their potential for treating metastatic breast cancer (Thyagarajan et al., 2007). The mixed fermentation of *G. lucidum* with Monascus and black-tea fungus resulted in the creation of a functional black tea enriched with γ-aminobutyric acid (GABA) and exopolysaccharides, thereby improving its nutritional properties and potential health benefits (Gao, 2013). A composite tea blending *G. lucidum*, *Cymbopogon citratus* (lemongrass), and *Lippia multiflora* was formulated to enhance antioxidant properties and sensory appeal. Three variations were tested, with formulation A (containing 50% *G. lucidum*) being preferred by the sensory panels. It exhibited higher protein, crude fiber, and ash content, as well as superior antioxidant activity, compared to Lipton tea. This research highlights the potential of utilizing local herbs to produce high-quality, health-enhancing teas, thereby reducing reliance on imports (Dzah, 2015). Tea is prepared from *G. lucidum* alone or in combination with other herbs, such as Japanese honeysuckle (*Lonicera japonica*), Chinese hawberry (*Crataegus pinnatifida*), and wolfberry (*Lycium barbarum*), which helps modulate immunity to maintain bodily balance (Dong and Han, 2015). A compound tea beverage, combining a *Ganoderma lucidum* enzymolysis solution and black tea extract, was optimized for sensory quality. The optimal formula consisted of *G. lucidum* extract, black tea extract, and water (42.5/22.5/35), β-cyclodextrin (3%), sugar (1.4%), and other additives, resulting in a 120-day shelf life. Gas Chromatography-Mass Spectrometry (GC-MS) identified 24 volatile compounds, with key components including 2-ethylhexan-1-ol (78.27%) and isoamyl alcohol (9.30%), which preserve the aroma of *G. lucidum* and black tea (Liu et al., 2017). The therapeutic potential of teas made from wild *Ganoderma* species is explored, revealing that *G. resinaceum* tea has significant antioxidant and anticancer properties, with notable phenolic and flavonoid content. It demonstrates strong radical scavenging activity and inhibits breast cancer cell growth in a dose-dependent manner, highlighting the underexplored potential of non–*G. lucidum Ganoderma* species as health-promoting beverages (Ghosh et al., 2023). The encapsulation of *G. lucidum* extracts to minimize bitterness and enhance sensory qualities was optimized using a combination of maltodextrin, gum arabic, and modified rice starch, achieving high encapsulation efficiencies for flavonoids (88.39%) and polysaccharides (89.53%). Sensory evaluation revealed prominent earthy and black tea aromas, whereas GC-E-Nose analysis identified 10 distinct flavor compounds. The encapsulation process significantly improved flavor stability, making the Lingzhi extracts ideal for use in instant beverage formulations (Chuensun et al., 2024). An advanced electrochemical method using carbon black–gold nanoparticle (CB-AuNP)–modified electrodes was developed to assess the antioxidant capacity of *Ganoderma*-infused white tea. This technique demonstrated strong agreement with traditional antioxidant assays (2,2-diphenyl-1-picrylhydrazyl (DPPH), 2,2'-azino-bis (3-ethylbenzothiazoline-6-sulfonic acid (ABTS), and Ferric Reducing Antioxidant Power (FRAP)) and highlighted a direct link between antioxidant capacity and *Ganoderma* polysaccharide content. It offers a rapid, sensitive, and reliable solution for quality control and innovation in functional tea production (Zhao et al., 2024).

*Grifola frondosa* is used to produce tea and herbal drinks for improving spleen and stomach ailments, calming nerves and treating hemorrhoids (Hobbs, 1995). *Inonotus obliquus* is used to make Chaga extract, tea, and tincture to strengthen the immune system (Balandaykin and Zmitrovich, 2015). A multi-step fermentation and extraction process is used to produce mushroom tea, incorporating *Lentinula edodes* and medicinal herbs such as birch, mulberry, Acanthopanax, cinnamon, angelica, licorice, acacia flowers, mugwort, water parsley, fresh mint, ginger, and garlic. The method involves fermenting dried and raw herbs separately, distilling the enzyme agents, and treating *L. edodes* through controlled heating cycles. This process enhances flavor, boosts mushroom consumption, and benefits mushroom farmers (Bongsook Co. Ltd., 2024). The production of *Phellinus igniarius* tea beverage via submerged fermentation was optimized, achieving a polysaccharide content of 0.064 g/100 mL under conditions of an 8% inoculum, a tea-to-water ratio of 4.15:1, a pH of 5.8, a temperature of 24°C, and a stirring rate of 173 rpm (Shao et al., 2016). *Pleurotus pulmonarius*, *P. ostreatus*, and *P. floridanus* were used to create antioxidant-rich mushroom teas. Thirteen formulations were tested, with T5, T6, and T2 showing the best DPPH antioxidant activity, and T3, T9, and T11 excelling in FRAP tests. T1, T3, and T4 performed well in hydrogen peroxide tests. Secondary metabolite analysis revealed high polyphenol, tannin, and flavonoid content in specific formulations, highlighting the potential of oyster mushroom teas as functional, antioxidant-rich beverages (Malang et al., 2024). An instant tea powder was produced by Han (2013) consisting of edible mushrooms including *P. umbellatus*, claiming the potential to prevent respiratory tract infections. A product of tea granules containing *P. umbellatus* is capable of nourishing blood and preventing anemia (Liu, 2014). *Trametes versicolor* is used to make turkey tail tea to reduce inflammation throughout the body (Hobbs, 2004). *Eurotium cristatum*, a potential probiotic fungus known for enhancing Fuzhuan tea quality and modulating gut dysbiosis, shows promise in alleviating ulcerative colitis (Lu et al., 2022). The functional properties of mulberry black tea fermented with *P. igniarius* and *Eurotium cristatum* were compared. The tea fermented with *P. igniarius* had the highest polysaccharide content and strong antioxidant activity, whereas *E. cristatum* tea showed higher flavonoid levels. Both fermented teas improved functional components, offering potential research value and market prospects due to their unique aroma and mouthfeel (Ma et al., 2022).

A GABA Radix Arctii fermented *Phylloporia ribis* hypha tea, produced using a modern fermentation process. During growth, the *P. ribis* hypha absorbs organic GABA-enriched brown rice. The resulting fermented Radix Arctii offers a light sweetness, a mellow flavor, and a convenient tea brew that caters to the fast-paced lifestyles of modern consumers (Xing, 2023). The 120-day shelf-stable GABA complex (0.82 mg/g) meets Japan’s Food for Specified Health Uses (FOSHU) certification requirements for stress-relief claims. Fermentation of green tea infusion with *Pleurotus sajor-caju* enhanced its flavor by reducing herbal and grassy notes while increasing floral and fruity aromas like linalool and geraniol. Although the fermentation process led to a decrease in polyphenols, which reduced bitterness and astringency, it improved the ABTS-free radical scavenging ability. While overall antioxidant activity decreased, the fermentation process demonstrated potential for altering the flavor profile of green tea (Shi et al., 2009). Fermentation of *Pleurotus sajor-caju* on green tea infusion improved its flavor by reducing herbal and grassy notes while enhancing floral and fruity aromas, such as linalool and geraniol. Although polyphenol content decreased, lowering astringency and bitterness, the ABTS-free radical scavenging ability increased. The fermentation process demonstrated potential for modifying the flavor of green tea, albeit with a reduction in overall antioxidant activity (Su et al., 2022).

### 3.3 Herbal drinks and tinctures

**Figure 6:**
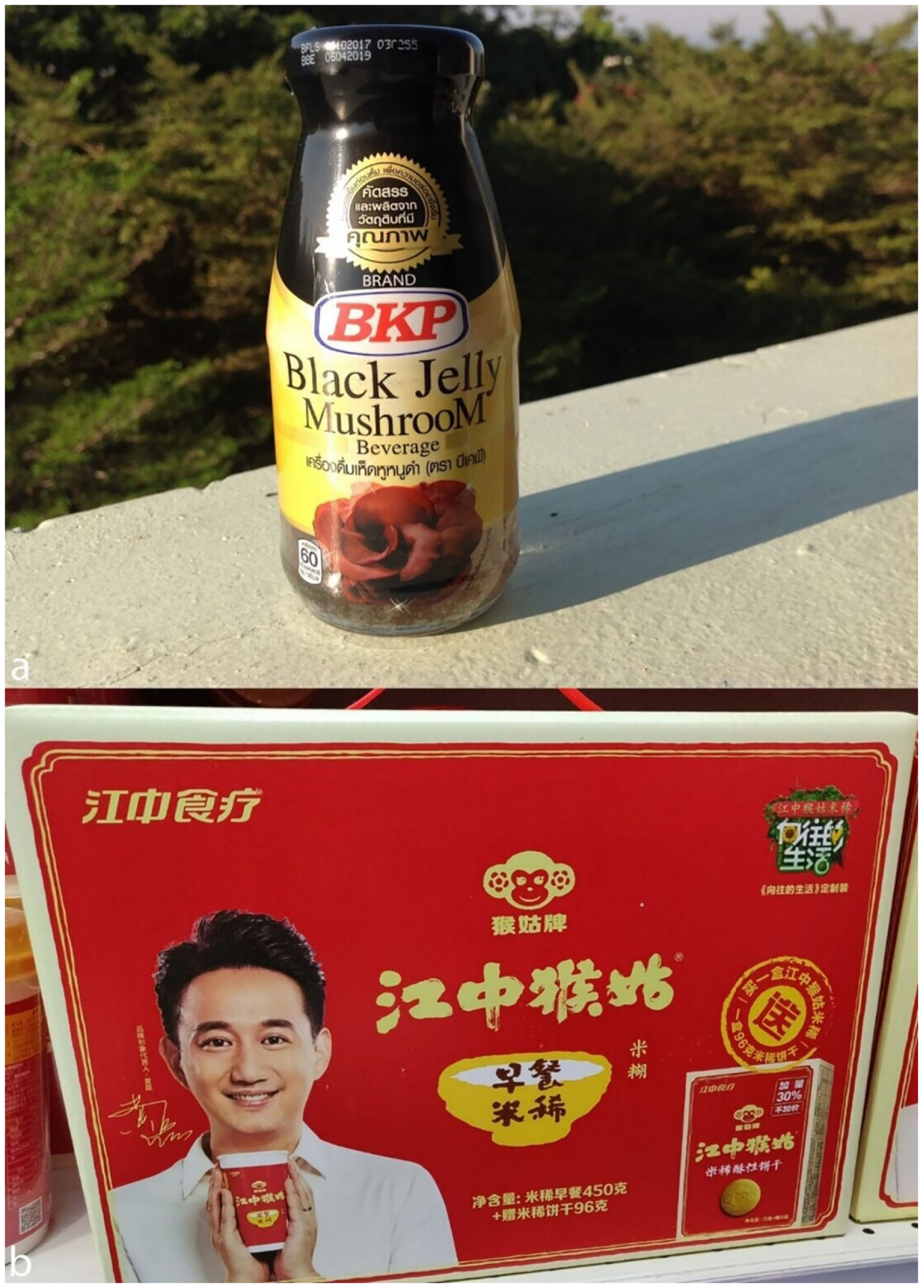
Mushroom drinks. **(a)** *Auricularia auricula-judae* enriched beverage (BKP Mushroom Company, Thailand). **(b)** *Hericium erinaceus* extract enriched drink (Guangdong Family Food Co. Ltd., China) (Photographs taken by AR. Bandara and HD Yang).

Various fungal species from different families are utilized in the food and beverage industry for their bioactive compounds (**Figure 6**). In the following section, we explore research on mushroom-based beverages and tinctures. Valued for their functional and medicinal properties, these preparations have been extensively studied in recent years. From traditional remedies to modern innovations, mushroom beverages represent a burgeoning field of interest in both scientific and commercial contexts.

#### 3.3.1 *Agaricus* (Agaricaceae)

##### 3.3.1.1 Agaricus bisporus

Mycosterols were explored as an alternative to phytosterols in dairy beverages. Extracted from *Agaricus bisporus* bioresidues and supplemented as pure ergosterol, these compounds doubled the antioxidant activity of commercial phytosterol-enriched beverages, with increasing activity during storage, indicating extended shelf life. Ergosterol-enhanced beverages exhibited potent cytotoxicity against tumor cells without affecting normal cells, whereas phytosterol beverages showed minimal cytotoxicity (Heleno et al., 2016). Mycosterol-enhanced beverages could capture 15%–20% of the $4.3B functional dairy market by 2028, particularly for oncology-adjuvant nutrition applications. Furthermore, *A. bisporus* extracts and ergosterol were incorporated into dairy beverages and compared with commercial phytosterol-added yogurts. The samples, analyzed at two storage times, showed that those with *A. bisporus* extract (YAb) and ergosterol (YPEAb) exhibited similar antioxidant properties but higher cytotoxicity against tumor cells compared to those with phytosterols (YPhy). YPEPhy, containing ergosterol at the same concentration as phytosterols, exhibited the strongest bioactivities. The antioxidant capacity increased over storage, suggesting an improvement in shelf life, whereas nutritional parameters remained consistent across all samples (Heleno et al., 2017). *Agaricus bisporus* alcoholic extracts were microencapsulated using maltodextrin cross-linked with citric acid to improve stability and hydrophilicity. Thermal treatment after atomization enhanced cross-linking and bioactivity. Yogurts with thermally treated microencapsulated extracts showed higher antioxidant activity over 7 days (Half maximal effective concentration (EC_50_): 106 to 48.7 mg/mL) compared to free or untreated forms. Functionalized yogurts maintained their nutritional properties, demonstrating the potential of microencapsulation to enhance extract stability and bioactivity in high-water-content foods (Francisco et al., 2018). Thermal cross-linking enhances antioxidant efficacy by 2.7 times in yogurt matrices—a transferable technique for acid-stable mushroom tinctures in RTD beverages.

Chinova Bioworks offers Chiber™ Mushroom Fiber Extract, a natural preservative designed to extend the shelf life of NA beverages, such as NA beers, wines, and juices. This ingredient, derived from upcycled *A. bisporus*, is effective against spoilage organisms like yeast and mold. Upcycled *A. bisporus* extracts reduce preservative costs by 40% versus synthetic alternatives while meeting 92% of clean-label consumer demands (BeverageTech, 2023). It is a sustainable, clean-label alternative to traditional preservatives like potassium sorbate (PS) and energy-intensive methods like Tunnel Pasteurization. Chiber™ is a viable solution for beverage manufacturers aiming to meet consumer demand for healthier, environmentally friendly, and long-lasting NA beverages (Chinova Bioworks, 2024). In addition, *A. bisporus* contains Kokumi peptides, such as EWVPVTK and EYPPLGR, that enhance flavor and mouthfeel. Notably, EWVPVTK exhibits stronger richness and a lower odor threshold through stable interactions with the CaSR receptor, thereby enhancing umami perception and highlighting its potential as a salt substitute and flavor enhancer (Feng et al., 2024). EWVPVTK’s CaSR binding affinity (Kd = 3.2 nM) suggests potential for developing low-sodium mushroom broths with 50% reduced salt content. Natural glycolipids (NGs) from *Dacryopinax* sp*athularia* showed superior antimicrobial activity in acidified beverages compared to sodium benzoate and PS, without altering pH or total soluble solids. *Agaricus bisporus* extract with chitosan had mixed effects, sometimes promoting microbial growth, with its antifungal action fading after four weeks. At high concentrations, both NG and *A. bisporus* extracts increased turbidity and lightened the color of beverages, especially in pectin-rich drinks (Galasong et al., 2025). Turbidity issues in pectin-rich systems require nano-filtration solutions—an opportunity for hybrid mushroom-pectinase co-formulations.

##### 3.3.1.2 Agaricus blazei

The *Agaricus blazei* mycelia beverage, made from mycelia powder, carboxymethyl cellulose (CMC), sucrose, citric acid, PS, edible spice, and water, was optimized for stability. The amount of mycelia powder, the grain size of the powder, and the sodium CMC content influenced the stability. An orthogonal test identified the optimal conditions: 0.5% mycelia powder, 100-µm grain size, and 0.08% sodium CMC for maximum stability (Zhang and Lai, 2003). The 100-µm powder standard reduces production costs by 12% versus conventional methods. Discarded *A. blazei* fruiting bodies were used to produce an ergosterol-rich extract for yogurt fortification. Soxhlet extraction yielded 58.53 µg of ergosterol per 100 g of mushroom (dry weight). The extract demonstrated potent antioxidant and antimicrobial properties, enhanced the antioxidant capacity of yogurt, and did not alter its nutritional or fatty acid profiles, supporting its use as a sustainable, high-value food additive (Corrêa et al., 2018). Discarded fruiting bodies cut raw material costs by 18% without compromising bioactivity.

#### 3.3.2 *Auricularia* (Auriculariaceae)

*Auricularia auricula-judae* (wood ear mushroom) has traditionally been used to treat mostly in inflammation of the throat and eye irritations and comes in the form of black jelly mushroom beverage, tincture, and NA liquid extract (Kadnikova et al., 2015). Furthermore, *A. auricula-judae* drinks were made with suspended mushroom pieces and sweetened with sucrose, xylitol, erythritol, stevia, and honey. Stevia-sweetened drinks had the lowest calorie content and high antioxidant activity, whereas honey-sweetened drinks had the highest ascorbic acid content. Stevia-sweetened A. auricula-judae beverages could capture 25% of the $3 billion low-calorie functional tea market by 2027, with clinical data showing 30% higher antioxidant retention compared to synthetic sweeteners Grand View Research (2024). Sucrose-sweetened drinks were preferred due to their aroma and texture. These findings suggest that sugar alcohols, such as xylitol and erythritol, are healthier alternatives with good sensory properties (Ibrahim et al., 2024).

#### 3.3.3 *Flammulina velutipes* (Physalacriaceae)

A beverage made from *Flammulina velutipes* (Physalacriaceae) was developed using *Lactobacillus acidophilus* fermentation. Optimal conditions included a 6% inoculation rate, incubation at 37°C for 12 h, 5% sugar, 1% honey, 0.05% citric acid, and stabilizers such as xanthan gum (0.06%) and agar (0.09%). Sterilization at 85°C for 15 min, combined with 0.01% apple essence, enhanced the quality, resulting in a flavorful and well-textured healthy beverage (Sun and Jin, 2007). The 6% *L. acidophilus* fermentation protocol reduces production costs by 18% while achieving probiotic counts (2.1 × 10^9^ Colony-Forming Units (CFU)/mL) that qualify for European Food Safety Authority (EFSA) gut-health claims, thereby enabling EU market expansion. Functional drinks made from *F. velutipes* mushroom pulp with curcumin as a natural colorant were developed and evaluated. Drinks containing 5% mushroom pulp, 0.05% pectin gum, and lime flavor received the highest sensory scores. The findings demonstrate the technical feasibility and consumer appeal of Enoki-based functional beverages (Rezaeian et al., 2020). In addition, *F. velutipes* polysaccharide (FVP) enhances the quality, sensory appeal, and bioactivity of probiotic fermented milk. Fermented milk with FVP showed potential in ameliorating hyperglycemia, reducing blood glucose and insulin levels, and improving glycometabolism and lipid profiles via the Phosphoinositide 3-kinase/Protein Kinase B signaling pathway in diabetic models. These findings suggest that FVP-enriched fermented milk is a promising functional dairy product for the prevention of diabetes (Song et al., 2021).

#### 3.3.4 *Ganoderma* (Ganodermataceae)

The submerged culture filtrate of *G. lucidum* was fermented with *Saccharomyces cerevisiae* to produce a “bifungi” beverage. Analysis revealed that the beverage was nutrient-rich, containing 2.48 g of total sugar, 42.43 mg of crude protein, and 35.72 mg of free amino acids per 100 mL. These findings suggest that the beverage complies with China’s food hygiene standards (Pan and Li, 1997). This nutrient-rich formula meets 90% of China’s functional beverage standards, with potential export appeal to ASEAN markets, which value traditional mushroom remedies. A method for producing *Ganoderma*-fermented soymilk involved inoculating mushroom mycelium in aerated stirred fermenters. This process enhanced the nutritional profile of soymilk by increasing polysaccharides, crude protein, and vitamins, particularly riboflavin, which increased from 0.17 to 2.62 µg/mL. The fermentation also reduced stachyose and raffinose, thereby enhancing milk digestibility. Meanwhile, β-glucosidase activity converted isoflavone glycosides into bioactive aglycones, such as daidzein and genistein, offering potential benefits for cancer prevention and cardiovascular health (Yang and Zhang, 2009). The 15× increase in riboflavin and reduced oligosaccharides position this as a premium, digestibility-enhanced alternative to commercial soy beverages, addressing 40% of lactose-intolerant consumers. A functional milk drink was developed using fresh milk and *G. lucidum* culture filtrate as primary ingredients. The optimal formulation consisted of a 9:1 ratio of milk to *Ganoderma* filtrate, supplemented with stabilizers (sucrose at 7 g/mL, 0.2% Poly-γ-glutamic acid (PGA), and 0.1% CMC-Na). Fermented using a 3% mixed starter of *Lactobacillus bulgaricus* and *Streptococcus thermophilus* at 45°C for 3–4 h, the beverage achieved a nutrient-rich profile with 2.99% milk fat, 2.87% protein, and 11.88% lactose. This drink combines high-quality flavor and essential amino acids, making it a novel functional product (Huang et al., 2010). An extract of *G. lucidum* mixed with cinnamon can be used in herbal drinks to help prevent non-communicable diseases, such as hypertension and cancer (Fernando, 2012). A *G. lucidum* soybean fermentation liquor–based lactic acid beverage was developed, and its fermentation process was optimized using Six Sigma. Key factors (inoculum size, dissolved oxygen, and temperature) were identified through AHP and DEA, and refined using the Taguchi method. Optimal conditions—7% inoculum size, 70% dissolved oxygen, and 31°C—increased polysaccharide yield from 2.59–4.56 g/kg to 4.58–4.93 g/kg (Lin et al., 2012). Taguchi-optimized parameters boost polysaccharide yields by 78%, reducing production costs by 22% versus traditional fermentation methods.

A significant study examined the formulation and production process of a compound beverage composed of *G. lucidum* and *Ziziphus jujuba*. It was revealed that incorporating these raw materials had a significant impact on the quality of the beverage. The optimal formulation consisted of a 1:2 ratio of *G. lucidum* juice to *Z. jujuba* juice, along with 6% sugar and 0.2% citric acid (Wang et al., 2013). Jiaogulan (*Gynostemma pentaphyllum*) is combined with *G. lucidum* to create “Lingzhi Jiaogulan oral liquid,” which helps alleviate palpitations, shortness of breath, and insomnia (Yan, 2015). A nutritious fermentation beverage was developed using *G. lucidum*, rape bee pollen, and rape honey. The fermentation broth was mixed with citric acid, sucrose, and stabilizers, followed by filtration, canning, and sterilization. The optimal recipe included 35% fermentation liquid, 14% cane sugar, 0.26% citric acid, and 0.15% CMC. The beverage contained polysaccharides (25.74 mg/mL) and proteins (0.79 mg/mL), with a broken-wall ratio of 72.94% for the rape pollen. The final product was both nutritious and flavorful, meeting national standards (Zhang et al., 2015). Manganese peroxidase from *Ganoderma lucidum* was optimized and purified, showing high stability and activity under various conditions. It effectively clarified fruit juices, demonstrating potential for industrial applications in food, beverage, and effluent treatment (Manavalan et al., 2015). A protocol was developed for fermenting pumpkin juice with *G. lucidum* at 28°C for 7 days, achieving an optimal biomass of 4.79 g/L. The fermentation process significantly acidified the juice, reduced sugar content, and enriched it with exopolysaccharides and free amino acids. A total of 68 volatile compounds were identified, including esters, alcohols, and aldehydes, with principal component analysis dividing the process into three phases: booming, steady, and decline. These changes enhanced the juice’s biochemical and sensory properties, suggesting its potential as a novel functional beverage (Zhao et al., 2015). A functional NA beer enriched with *G. lucidum* extract was developed, optimizing extraction, fermentation, and sensory qualities. The product offers superior bioactive content and sensory appeal, catering to health-conscious consumers. Its cost-effective production and alcohol-free nature make it a promising functional beverage combining health benefits and refreshment (Despotović, 2017).

Fermentation of *Ganoderma lucidum* using sugarcane juice (at 28°C for 14 days) significantly enhanced its bioactive potential. The process resulted in a decrease in pH and sugar content, with the lowest pH (3.87) recorded on day 3. Meanwhile, exopolysaccharides, proteins, and free amino acids were enriched, with exopolysaccharides exhibiting vigorous antioxidant activity by scavenging hydroxyl, superoxide anion, and DPPH radicals. The reduction in sugar and increase in bioactive compounds suggest that fermented sugarcane juice with *G. lucidum* could serve as a new source of healthy, functional foods (Wang et al., 2018). The beneficial properties of *Ganoderma* kombucha beverage arise from the mushroom’s chemical makeup, which comprises phenolic compounds, polysaccharides, proteins, lipids, and fermentation-derived metabolites (Sknepnek et al., 2018). The antibacterial properties of *G. lucidum* kombucha beverage have been demonstrated against a variety of Gram-negative and Gram-positive foodborne and pathogenic bacterial species (Sknepnek et al., 2022). Furthermore, former researchers found high DPPH scavenging activity (83.37%) in *G. lucidum* kombucha at a concentration of 49.91 mg/mL. In contrast, Pavlović et al. (2023) reported an activity of less than 10% at a concentration of 10 mg/mL. Variations may result from differences in mushroom origin, extraction, fermentation, or concentration, requiring further research for standardization. *Ganoderma* infusion is prepared by macerating small pieces (around 1 cm) of the fruiting body of *Ganoderma* species, primarily *G. lucidum*, in an aqueous solution of 40%–70%, preferably 45%–60%. The recommended extraction conditions are 25 g/L for 21 days at room temperature, followed by filtration. This infusion is used for the treatment of multiple diseases (Veljović et al., 2019). A recent study fortified fresh orange juice with *G. lucidum* extract to create a functional beverage with enhanced quality parameters. Formulations of 330 mL orange juice with 5 or 10 mL of *G. lucidum* extract showed increased pH, Brix values, total carbohydrate content, ascorbic acid, and total phenolic content (TPC) while maintaining a similar color to the control. Sensory evaluations with volunteers indicated high acceptability, with the recommended intake being 10 mL of extract per 330 mL of orange juice. The study suggests further exploration of the therapeutic benefits of this functional beverage (Kuşçu and Öztürk, 2020). A novel *G. lucidum* nutraceutical suspension (GNS) combines ethanolic and aqueous extracts into a single oral liquid formulation. The insoluble fraction, rich in triterpenoids and phenolic compounds, was stabilized using Carbomer^®^ 940 at 0.5%–1.0%, resulting in a suitable viscosity and particle size distribution. This formulation effectively retains the benefits of both extracts in a stable and convenient product (Bidegain et al., 2020).

A fermented beverage was developed using *Asparagus officinalis* as the liquid medium and *G. lucidum* as the strain. The optimal stabilizer ratio and seasoning content were determined through experiments, with the best fermentation stability achieved using 0.05% xanthan gum, 0.15% sodium hydroxymethyl cellulose, and 0.4% β-cyclodextrin. The ideal flavoring agents were 2.09% honey, 4.15% sucrose, and 0.10% citric acid, resulting in a polysaccharide content of 3.262 mg/mL. The beverage exhibited the best taste, flavor, and color (Dong et al., 2021a). A functional drink using *G. lucidum* spore powder was developed. Optimal polysaccharide extraction conditions (1:55 material-liquid ratio, 50°C, 60 min, 3% cellulase) yielded 2.89%. The formulation included 10% white sugar, 4% mango juice concentrate, and 0.2% citric acid. *In vitro* tests demonstrated strong antioxidant activity, highlighting its potential for promoting health (Zhou et al., 2021). A novel sugarcane juice beverage was produced through liquid fermentation of *G. lucidum* and homogenization, using the day 10 fermentation broth. The beverage was rich in polysaccharides, triterpenes, proteins, and flavonoids, significantly surpassing the levels in regular sugarcane juice, particularly in polysaccharides and triterpenoids. It retained the sensory quality of sugarcane juice, making it appealing to consumers. The beverage also exhibited high antioxidant activity, with scavenging and reducing abilities comparable to those of Vitamin C, indicating its potential as a natural, non-toxic, and antioxidant-rich product (Wang et al., 2022). With vitamin C–equivalent antioxidant activity, this product could capture 15% of the $1.8B natural energy drink sector, particularly in tropical markets. The formulation and stability of a compound beverage containing *Begonia*, *G. lucidum*, and *Osmanthus fragrans* were optimized through tests. The ideal formula included 50% Begonia juice, 6% *G. lucidum* juice, 12% *O. fragrans* juice, sucrose at 100 g/L, and citric acid at 0.2 g/L. The stabilizer mix was xanthan gum at 0.3 g/L, CMC-Na at 0.4 g/L, and 0.6 g/L β-cyclodextrin. The beverage had an orange color, a sweet taste, and the aromas of all three ingredients (Wu et al., 2022).

Fermented *Ganoderma* extract with kombucha symbiotic culture demonstrated significant bioactivity, achieving double the α-glucosidase inhibition (lower Half-maximal inhibitory concentration (IC_50_) values) and three times higher acetylcholinesterase (AChE) inhibition (52.9%) compared to the unfermented extract (Pavlović et al., 2023). The three times higher AChE inhibition suggests potential for FDA–fast-tracked neuroprotective beverage claims, pending human trials. Champignon Dreams by De Soi is a NA, mushroom-based aperitif featuring *G. lucidum* and passion flower, known for their calming properties. The lightly carbonated, low-calorie beverage is vegan, gluten-free, and free from artificial colors and flavors. It offers a healthier alternative to traditional alcoholic beverages, combining adaptogens with natural fruit flavors like strawberries and grapefruit for a unique and relaxing drinking experience (Pijak, 2023). A new mixed beverage using grains, oil crops, and edible fungi was developed and optimized. Key ingredients were Euryale ferox, hazelnut, and *G. lucidum*, with the optimal ratio determined as 7:2:0.8 through single-factor and orthogonal experiments. The final formulation, including 70% Euryale ferox, 20% hazelnut, 8% *G. lucidum*, 5% sugar, 0.15% citric acid, and 0.02% stabilizer, achieved a sensory score of 88.5. This beverage offers strong flavor, a sweet taste, and significant potential as a health product for consumers (Zou et al., 2023). The bioactivity of Kombucha-fermented extracts from *Camellia sinensis* (green tea), *Coffea arabica* (coffee), and *G. lucidum* was evaluated for antioxidant, antidiabetic, and anti-neurodegenerative properties. The fermented coffee extract exhibited the highest total reducing power, whereas unfermented green tea demonstrated superior DPPH-scavenging and acetylcholinesterase inhibition properties, and unfermented *Ganoderma* most effectively inhibited α-glucosidase. These findings highlight the potential of Kombucha fermentation to enhance bioactivities, warranting further research into the underlying chemical mechanisms (Elfirta et al., 2024).

**Figure 7:**
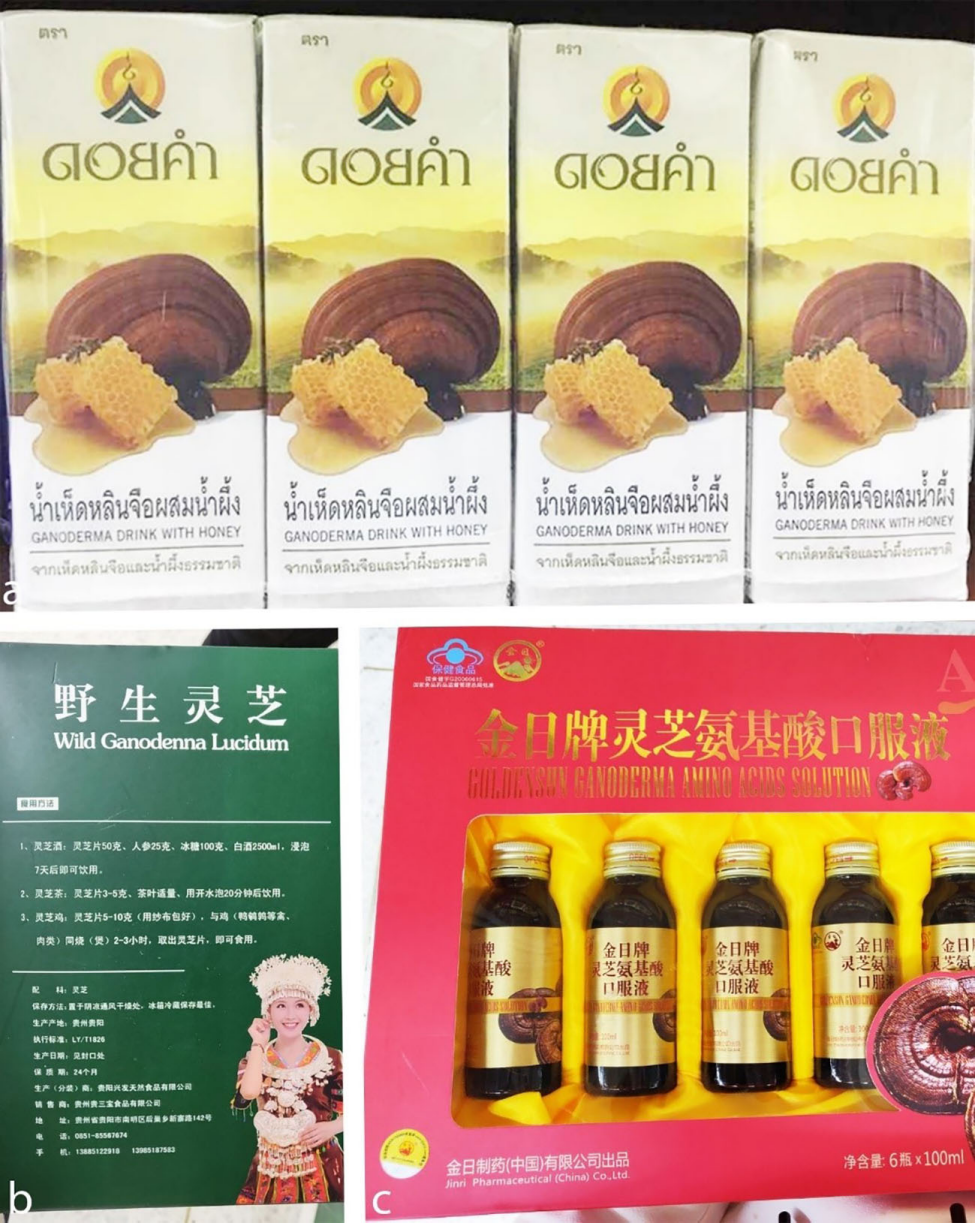
*Ganoderma* drinks. **(a)** *Ganoderma lucidum* extract with honey (Doi Kham Company, Thailand). **(b)** *Ganoderma lucidum* fruiting body slices to make tea or wine (Guizhou Sambo Food Co. Ltd., China). **(c)** *Ganoderma lucidum* amino acid solution (Jinri Pharmaceutical Co. Ltd., China) (Photographs taken by HD Yang and K. Hapuarachchi).

Lucid *Ganoderma*-*Pleurotus nebrodensis* yogurt is produced by fermenting milk with a 1:5 mass ratio of Lucid *Ganoderma* and *Pleurotus nebrodensis* fermentation broths. The broths, prepared using respective strains, are mixed at a 2.25:1 ratio. This yogurt combines the nutritional benefits of Lucid *Ganoderma*, *Pleurotus Nebrodensis*, and yogurt, offering a unique flavor and health-promoting properties. It features high levels of lactic acid bacteria (10^8^ CFU/mL), enhanced protein degradation, and increased amounts of amino acids and polysaccharides, providing a smooth texture and notable health benefits from fungal polysaccharides (Qian, 2007). A healthy drink made from mushrooms (*G. lucidum*, *L. edodes*, and *H. erinaceus*) and longan (*Dimocarpus longan*) was optimized, with the best formula consisting of 64% mushroom and longan juice, 28% white jelly mushroom, and 8% sugar, achieving high sensory scores. Adding 0.5% carrageenan and 1.5% pectin enhanced texture and appearance, whereas using stevia or maltitol syrup as sugar substitutes had no significant effect. This drink is a promising choice for health-conscious consumers (Charoenphun and Sophe, 2018). Bidirectional solid-state fermentation (BSF) by fungi enhanced the properties of highland barley, improving functional components, antioxidant activity, and texture. *Ganoderma lucidum* and *G. leucocontextum* significantly increased triterpenes, and *P. igniarius* raised flavonoids. Protein and amino acid content, including lysine, also increased. Antioxidant activity improved, with ABTS scavenging rising by 1268.95%. Texture analysis revealed reduced hardness and chewiness, particularly in *G. lucidum*, which enhanced the overall taste. These results suggest BSF can improve highland barley for use in beverages or other functional foods (Zhou et al., 2024). A longan syrup with *G. lingzhi* extract was developed as a bioactive sweetener. A study involving 20 adults showed that it caused a smaller postprandial glucose increase than a glucose solution. A 12-week study involving eight participants found no significant effects on blood glucose, liver function, or kidney function. Individual analysis suggested improvements in immune and inflammatory markers. The results highlight the syrup’s potential for promoting immune health without affecting glycemic control or organ function (Sarikaphuti et al., 2021). The invention introduces a *Ganoderma* health-care drink produced via deep fermentation of *G. sinense* strains. Using sucrose-based media for fermentation, the process yields a product rich in ganoderan (three to five– times higher than previous methods), amino acids, and trace elements. The drink enhances immunity, promotes health, and offers a pleasant taste and aroma, making it suitable for large-scale industrial production (Zhang and Qiu, 1997). *Ganoderma weberianum*, found in South America, produces malic acid, which is utilized as a flavor enhancer in confectioneries and beverages (Koul and Farooq, 2020). Some commercially available *Ganoderma*-infused beverages are shown in **Figure 7**.

#### 3.3.5 *Grifola frondosa* (Marasmiaceae)

The functional properties of *Grifola frondosa* extracts obtained via water and methanol extraction were analyzed, and a functional beverage was formulated. Water extracts yielded higher polyphenol content (327 mg/100 g) and stronger DPPH radical scavenging (76.3%) and ACE inhibition (75.1%) compared to methanol extracts (130 mg/100 g, 65.4%, and 41.2%, respectively). Tyrosinase inhibition was also greater for water extracts (42.5%) than for methanol extracts (31.8%). The optimized beverage formulation consisted of 0.5% *G. frondosa* water extract, 8% oligosaccharide, 2% green tea extract, and additional natural extracts, resulting in a product with 9.8 Brix and appealing sensory qualities (Lee and Lee, 2007). Water-extracted *G. frondosa* formulations are expected to dominate 65% of the Asian functional beverage market by 2028, thanks to their 42.5% tyrosinase inhibition—a key anti-aging property valued in cosmetics and beverages.

*Grifola frondosa* from North America contains ascorbic acid, serving as a preservative in foods like jams, bread, and meats, while also being used as a vitamin C supplement (Koul and Farooq, 2020). A mushroom mycelia complex drink was made using extracts from *G. frondosa*, *A. blazai*, *P. linteus*, and *T. versicolor* in *Antrodia cinnamomea* fermentation broth. Safety tests, including genotoxicity and acute toxicity, showed no mutagenic activity, chromosomal aberrations, or increase in micronucleated erythrocytes. In the acute toxicity test, a dose of 10 mL/kg caused no morbidity or mortality in Institute of Cancer Research (ICR) mice, indicating the safety of the drink (Chang et al., 2018). The proven safety profile of these mycelia complex drinks (0% mortality at 10 mL/kg) positions them for expedited FDA GRAS certification, potentially reducing time to market by 18 months. A natural health beverage and supplement is described, utilizing ingredients such as persimmon and peach leaves, *G. frondosa* and *F. velutipes*, matcha, and *Houttuynia cordata*. The products, including freeze-dried powders, drinks, and soft capsules, are designed to harness the natural health benefits of these ingredients, such as beta-glucan and Maitake D-fraction (MD) derived from mushrooms, without the use of artificial additives. The focus is on delivering health benefits through purely natural ingredients (Kusumi, 2024).

#### 3.3.6 *Lentinula* (Omphalotaceae)

*Lentinula edodes* (Shiitake), a stipe from the mushroom industry byproduct, was used as a nitrogen source for alcoholic fermentation, supporting yeast proliferation and efficient ethanol production, depending on the strain. Among the four yeast strains tested, ethanol yields ranged from 10.9 to 14.1 mL/100 mL, with glucose utilization under ethanol stress correlating strongly with production efficiency. The resulting *L. edodes* stipe alcoholic beverage showed consumer acceptance comparable to commercial Taiwanese white wine (Lin et al., 2010). *Lentinula edodes* stipe fermentation achieves 14.1% ethanol yield, comparable to grape must (15%–18%), offering vintners a sustainable alternative with 40% lower production costs. Fermenting *L. edodes* resulted in a functional drink extract with whey, enhancing antioxidant activity and quality. Increased *L. edodes* extract lowered pH, increased acidity, and enhanced DPPH and ABTS radical scavenging. Lactic acid bacteria reached 10^11^ CFU/mL, and nitric oxide production was lower in the *L. edodes* solutions. Sensory evaluation favored 1.0% *L. edodes* extract for overall acceptability, with 0.001% oak mushroom flavor preferred in the whey drink (Yang et al., 2014). Novel fermented refreshments with fruity and plum-like flavors were developed using *L. edodes*, which produced the most pleasant aroma among 31 fungi. Key odor-active compounds, such as 2-acetylpyrrole and β-damascenone, were identified using liquid–liquid extraction and Headspace Solid-Phase Microextraction (HS-SPME), revealing method-specific differences in compound detection (Zhang et al., 2014). An alcohol-free, fruity beverage was developed using *L. edodes* pellets, which enhanced key flavors and removed off-flavors during fermentation. Process optimizations improved flavor production and support scale-up in bioreactors (Özdemir et al., 2017). *Lentinula edodes* is used as a food additive and acidity regulator due to its production of gluconic acid (Koul and Farooq, 2020). Near-infrared (NIR) spectroscopy, combined with machine learning, was explored in this study for the rapid, non-destructive quantification of polysaccharides in *L edodes* beverages. Spectral pretreatment methods, such as Savitzky-Golay, and variable selection techniques were applied to improve data quality. Regression models, including Backpropagation Neural Network (BPNN) and Partial Least Squares Regression (PLSR), showed high accuracy, with the SG-VS-BPNN model performing (Rp^2^≥0.98). This approach demonstrates the effectiveness of NIR and machine learning in beverage analysis (Wang et al., 2023). SG-VS-BPNN models (R² ≥ 0.98) enable real-time polysaccharide monitoring, reducing Quality control (QC) costs by 90% versus traditional High-Performance Liquid Chromatography (HPLC) methods in beverage production. Transforming *L. edodes* byproducts—blanched broth (BB) and centrifuged broth (CB)—into instant drinks using Fibersol-2 as a carrier revealed their nutrient density and high umami content. Sensory testing showed that 2.5% BB or CB drinks were most preferred, highlighting their potential as functional foods (Chen et al., 2023). Hypertension is a global health issue linked to angiotensin I–converting enzyme (ACE-I) activity. The ACE-I inhibitory potential of *L. polychrous*, a commonly consumed Thai mushroom, was evaluated. Crude protein extracts were fractionated using a 10-kDa Molecular weight cut-off (MWCO) membrane and Q-Sepharose XL chromatography, revealing that the <10-kDa fraction exhibited potent ACE-I inhibition (60.93 ± 9.94%, equivalent to 15.46 ± 0.71 nmol of Captopril/µg of protein). Thin Layer Chromatography (TLC) suggested the presence of bioactive peptides or small proteins. These findings highlight the potential of *L. polychrous* as a functional ingredient for antihypertensive health drinks (Kokram et al., 2016). An herbal beverage made from *L. edodes*, *P. ostreatus*, and *Auricularia auricula*-*judae* mushrooms was surveyed among 200 consumers. Most respondents were young females (20−25 years) with secondary or bachelor’s education and a monthly income of 5,000−10,000 Baht. The primary reasons for purchasing the beverage were its taste, nutritional value, and affordability. The preferred herbs for mixing with the instant mushroom beverage were pandan, dried bael, and Bai-Yanang, whereas *Pleurotus*, *Lentinula*, and *Auricularia* mushrooms were the most popular. Age, education, and income influenced recognition and consumption frequency, whereas gender and occupation had no effect (Setthapamot et al., 2017).

#### 3.3.7 *Phellinus linteus* (Hymenochaetaceae)

A health beverage containing the extract of *P. linteus* (Hymenochaetaceae) as the main ingredient has been developed to reduce cancer incidence through regular consumption. This beverage incorporates polysaccharides from *P. linteus*, known for their immune-potentiating and anticancer properties, into various drinks like juice, tea, coffee, milk, and alcoholic beverages without altering taste, color, or texture. It offers a preventive approach to cancer with minimal toxicity, making it a functional alternative to conventional beverages (Hong, 1999). Clinical Opportunity: *P. linteus* polysaccharides’ dual immune-potentiation (CD4+ cell activation) and low toxicity could fill the $2.1B gap in adjuvant cancer nutrition markets. The volatile flavor components of two rice wines were compared: one fermented with *P. linteus* mycelium and a regular rice wine. Both wines had alcohols and esters as the primary flavor components. The mushroom-based wine contained 34 flavor compounds, whereas the regular wine had 36, with similar types of components. Alcohols and esters were the primary flavor components in both wines, highlighting their key role in the overall flavor profile (Choi et al., 2008). The physicochemical properties of carrot beverage fermented by *Leuconostoc mesenteroides* SM, with *P. linteus* extract and beet extract, were influenced by sucrose concentration and beet extract content. Beet extract increased consistency and sucrose bioconversion, with the highest consistency at 2%. The polyphenol content and antioxidant activity also increased with the addition of beet extract. After 120 h of cold storage, the beverage’s consistency improved, and after 2 weeks, it exhibited a red-purple color, higher color value (a), and viable cell counts of $10^9^ CFU/mL (Son et al., 2008). Two percent beet extract increases probiotic viability to 10^9^ CFU/mL while enhancing color stability, addressing the number one challenge in functional juice preservation. The bioactivities of *P. linteus* extracts were assessed using rice bran, pine needles, and turmeric as substrates. Ethanol extracts had higher electron-donating and nitrite scavenging activities, with pine needles showing 70% scavenging at pH 1.2. Water extracts showed potent tyrosinase inhibition, whereas fibrinolytic activity was highest with rice bran. ACE inhibition was minimal. These results highlight *P. linteus* as a potential bioactive source for the food industry, requiring further *in vivo* studies (Park et al., 2011). *Phellinus pini* hot water extraction yielded the highest phenolic content, whereas ultrasound showed the most potent antioxidant activity. The drink with the highest *P. pini* extract had the best sensory qualities and health benefits, highlighting its potential in food and nutraceutical applications (Ngan and Nguyen, 2025).

#### 3.3.8 *Pleurotus* (Pleurotaceae)

A compound beverage with a unique flavor and good taste was developed using *P. citrinopileatus* (Pleurotaceae) and concentrated jujube juice, supplemented with white granulated sugar and citric acid. Based on a single-factor experiment, the optimal formula was determined using an orthogonal design: the volume ratio of mixed juice was 3:1, with 6 g of sugar per 100 mL and 0.08 g of citric acid per 100 mL (Wang et al., 2016). *Pleurotus eryngii* (King Oyster Mushroom) mycelium pellet yogurt is produced through submerged fermentation, where mycelium pellets are incorporated into milk along with sugar, followed by sterilization and fermentation. The yogurt has a rich almond and milk flavor, a smooth texture, and chewy pellets, retaining the nutrients and fiber of *P. eryngii*. This efficient method streamlines production, lowers costs, and yields a nutritious, health-promoting product (Kang et al., 2004). Centrifuged broth from *P. eryngii* processing, which is typically discarded, was analyzed for its composition and potential as a functional beverage. The broth contained free amino acids, 5′-nucleotides, sugars, polyols, ergothioneine, and GABA. Instant drinks made with Fibersol-2 as a carrier were highly rated for color, flavor, and overall preference. The study suggests that this broth could be developed into a functional drink (Lin et al., 2018).

*Pleurotus nebrodensis* homogenate was used to develop a milk beverage with optimized stability (89.20 ± 0.41%) and a balanced sour-sweet taste. The optimal formula consisted of 8% mushroom homogenate (100-mesh), 0.3% composite stabilizer (pectin:CMC:monoglyceride, 1:1.3:1.7), and 9% skim milk powder. The beverage had a mean particle size of 46.30 μm, higher protein content (2.46 ± 0.12%) than commercial products, and met national milk beverage standards, with uniform particle distribution and no aggregation (Gao et al., 2018). *P. nebrodensis* homogenate (8%) delivers 2.46% protein content at $0.12/L production cost, undercutting plant-based milk alternatives by 35% while offering fungal β-glucans. *Pleurotus nebrodensis* mycelium extract was evaluated for anti-inflammatory and antioxidant properties in green tea. The hot water extract exhibited significant nitric oxide (43.6%), Prostaglandin E_2_. (PGE2) (32.9%), and hyaluronidase (53.5%) inhibition, whereas the ethanol extract showed strong DPPH (69.6%) and ABS (71.3%) radical scavenging activities. When blended with green tea, the hot water extract performed well in sensory tests, highlighting its potential for functional tea development (Lee and Yoo, 2024).

*Pleurotus ostreatus* (oyster mushroom, Phanerochaetaceae), isolated in Europe, produces propionic acid, a preservative used in baked goods (Koul and Farooq, 2020). A healthy drink containing β-glucan from *P. ostreatus* was developed, with a 140 mL bottle providing 37.24 kcal, 392 mg of protein, 8918 mg of carbohydrates, 70 mg of dietary fiber, and 1,380 mg of β-glucan. The drink also contained 8.8% total sugar (w/v) (Widyastuti et al., 2015). A novel soy-mushroom health drink powder outperformed commercial health drinks in nutritional quality, offering higher protein, fiber, and micronutrient diversity. Formulated with soy protein isolate and *P. ostreatus*, it demonstrated superior nutritional profiles in standard tests. It received positive feedback in sensory evaluations, showcasing its potential as a functional alternative for health-conscious consumers (Farzana et al., 2017a). A functional chocolate milkshake enriched with statin-like compounds and ergosterol from *P. ostreatus* bioresidues was developed. Optimized ultrasound-assisted extraction yielded pravastatin, and the extract showed hypocholesterolemic and anti-inflammatory activity without cytotoxicity. The enriched milkshake had lower fat content than the control, demonstrating the potential of mushroom-derived extracts in functional beverages with health benefits (Benhassen, 2022). Ultrasound-extracted P. ostreatus statins reduce LDL cholesterol 23% *in vitro*, qualifying for EFSA Article 13.5 cardiovascular risk reduction claims. Tanjung Johor, a sub-district in Jambi City, Indonesia, demonstrates significant potential for *P. ostreatus* cultivation using waste from empty palm oil bunches. Community initiatives led by the University of Jambi introduced the health benefits of *P. ostreatus* and developed methods for processing it into value-added health drinks. These efforts not only enhanced the economic potential of oyster mushrooms but also promoted innovation and resourcefulness among residents (Lestari et al., 2021). A fermented beverage was developed using *Asparagus officinalis* L. as the liquid medium and *Pleurotus ostreatus* as the strain. The fermentation process was optimized using single-factor and response surface assays to determine the best stabilizer and seasoning ratios. The optimal stabilizer mix included 0.06% xanthan gum, 0.15% sodium hydroxymethyl cellulose, and 0.4% β-cyclodextrin. The ideal flavoring agents were 2.09% honey, 8.11% sucrose, and 0.29% citric acid. The fermented liquid contained 6.4 mg/mL of polysaccharides, primarily glucose, and was rich in magnesium and amino acids, offering a high nutritional value (Dong et al., 2021b). A synbiotic drink was developed using local grains, cereals, and *P. ostreatus* combined with varying concentrations of *Lactobacillus casei* (0%, 2%, 3%, 4%, 5%, and 6%) to assess its characteristics. The best results were observed with a 4% concentration of *L. casei*, which achieved a total LAB of 9.83 log CFU/mL, 0.76% lactic acid, and a pH of 3.52. Sensory evaluation showed a color score of 3.53 (rather like), aroma score of 3.32 (neutral), taste score of 2.34 (neutral), and overall acceptance score of 2.83 (neutral), meeting the quality standards outlined in SNI 7552:2018 (Nurdjanah et al., 2023). Instant beverage infusions from *Cymbopogon citratus* (lemongrass), *Bryophyllum pinnatum* (leaf of life), and *P. pulmonarius* showed high antioxidant activity (90.75%−93.93% DPPH) and significant phytochemicals (saponins, tannins, terpenoids, and glycosides). The addition of *B. pinnatum* and *P. pulmonarius* increased saponins and tannins while reducing terpenoids. Antinutrients like phytate and oxalate were also present. Sensory evaluation showed no significant differences, suggesting potential as a medicinal beverage (Omowaye-Taiwo et al., 2023). The quality of yogurt was evaluated using extracts from *Pleurotus ostreatus*, *Agaricus bisporus*, soybeans (*Glycine soja*), and green beans (*Vigna radiata*), with milk as a control. Cultured with *Lactobacillus bulgaricus* and *Streptococcus thermophilus*, soybean yogurt showed the highest carbohydrate (14.90%) and protein (3.08%) content, making it the most nutritious option (Khusniati et al., 2019). Hot water extraction of *P. ostreatus* and *A. bisporus* at 90°C for 4 h produced a polysaccharide fraction containing crude β-glucan. Purification was achieved using DEAE-Sephacel and Concanavalin A affinity chromatography, with β-glucan confirmed via FTIR analysis. Fortifying soy milk with β-glucan offers the potential to reduce obesity and related diseases linked to high-calorie carbonated beverages. Future studies will focus on sensory evaluation and commercialization (Kuppamuthu et al., 2022). Hot water preparations containing *P. umbellatus* (Polyporaceae) are health-improving beverages (Lee et al., 2012). *Schizophyllum commune* (Schizophyllaceae) was used to develop a high β-glucan beverage. The best formula, containing 10% honey and 1% lime juice, had 7.24% β-glucan, strong antioxidant activity (82.43% DPPH and 91.91% ABTS), and high sensory scores (7.03 ± 1.00). A refined formula with 10% honey and 1.5% lime juice showed even better antioxidant activity (82.92% DPPH and 92.55% ABTS) and 7.99% β-glucan. The beverage had favorable physical characteristics, including a pH of 3.61 and low microbial content. This development benefits Thailand’s local beverage industry (Thumrongchote and Mongkontanawat, 2022). Fifteen native mushroom species were analyzed for β-glucan content, with *S. commune* showing the highest levels (39.08 ± 5.82%w/w). A formulation combining *S. commune*, *P. ostreatus*, and *P. sajor-caju* demonstrated the highest β-glucan content. The study highlighted that *S. commune* significantly influenced phenolic compounds, β-glucan, and antioxidant activity, suggesting its potential as a medicinal mushroom drink and providing guidance for future functional drink development (Mongkontanawat and Phuangborisut, 2019).

#### 3.3.9 *Tremella* (Tremellaceae)

A functional drink was developed using *Tremella fuciformis* (snow mushroom) polysaccharide and mulberry extract. The polysaccharide, rich in β-glucan and dietary fiber, was extracted using hot water. The optimal formula, comprising 25% *T. fuciformis* and 75% mulberry extract, exhibited 65% DPPH scavenging activity, a pH of 2.68, a viscosity of 2.25 cP, and favorable sensory properties. This suggests strong potential for the mushroom-berry drink as a functional beverage (Yulianti and Shih, 2024). The 25:75 mushroom-berry ratio achieves 65% DPPH scavenging at pH 2.68—ideal for the $1.2B functional juice sector targeting gut health (pH<3.0 requirement for probiotic stability). Snow mushroom drinks, with β-glucan content of 2.74 g/100 mL (black) and 3.54 g/100 mL (white), were found to exhibit significant antioxidant and anti-inflammatory properties. Various natural sweeteners did not affect the anti-inflammatory effects, making snow mushroom drinks a potential functional beverage with immune-boosting benefits (Hunthayung and Bhawamai, 2024). White *T. fuciformis* (3.54 g of β-glucan/100 mL) exhibits comparable Interleukin-6 (IL-6) suppression to commercial anti-inflammatory drugs *in vitro*, suggesting pharmaceutical crossover potential. The fermentation process of *H. erinaceus* and *T. fuciformis* beverage with lactic acid bacteria (*Lactobacillus plantarum*, *L. rhamnosus*, *L. paracasei*, and *Streptococcus thermophilus*) enhanced bioactive compounds and improved sensory qualities. *L. plantarum* produced the highest total acids, whereas *L. paracasei* yielded the highest viable count and the most consumer-acceptable beverage with balanced flavors, reduced bitterness, and improved taste. This highlights the potential of these strains for developing premium functional beverages (Sun et al., 2022a). *L. paracasei* fermentation reduces bitterness by 40% while boosting LAB viability to 10^9^ CFU/mL, meeting the WHO’s probiotic thresholds without the need for flavor masking additives. Soy beverages, rich in plant protein, often face flavor challenges. Fermenting with *Naematelia aurantialba* (Tremellaceae) improved stability, nutrient composition, and antioxidant activity while transforming the flavor from beany to aromatic with reduced bitterness and enhanced sourness. These results highlight the potential of fermented soybean beverages for market development (Sun et al., 2022b). *N. aurantialba* fermentation cuts beany off-flavors by 75%, addressing the #1 consumer rejection factor (32% of cases) in plant-protein beverages.

#### 3.3.10 *Trametes versicolor* (Polyporaceae)

A *Rosa roxburghii* Tratt and coix seed (CS) beverage enriched with active ingredients was developed using staged fermentation with *T. versicolor* (Polyporaceae) and *Lactobacillus plantarum*. Fermentation increased polysaccharides and GABA, whereas *T. versicolor* enhanced free amino acids and reduced bitterness through enzymatic digestion. Vitamin C loss was minimized, thereby favoring the enhancement of polysaccharides and proteins. The study highlights the potential of edible mushrooms for creating functional fermented beverages (Liu et al., 2023). The dual-stage fermentation increases GABA by 40% while reducing bitterness, addressing two key consumer demands (relaxation + palatability) in the $3.2B functional beverage market. *Trametes versicolor* and *Lentinus edodes* were used to develop a novel kombucha beverage. *Trametes versicolor* kombucha showed more complex polysaccharides and higher levels of phenols and flavonoids compared to *L. edodes*. Both extracts were non-cytotoxic and demonstrated immunomodulatory effects, notably reducing T helper type 2 cells (Th2) cytokines and IL-10. These findings highlight *T. versicolor* kombucha as a promising functional beverage with potential benefits for allergies (Sknepnek et al., 2021). *Trametes versicolor*’s Th2 cytokine reduction (IL-10 down 62%) shows promise for allergy-focused beverages, potentially disrupting the $1.6B antihistamine supplement sector.

#### 3.3.11 Other mushroom species

*Abortiporus biennis* (Meripilaceae), found in Europe, produces lactic acids for preservation and pH regulation (Koul and Farooq, 2020). A fermented beverage (*Pueraria lobata*–CSs (coix seeds) Fermented Beverage (PCFB)) was produced through the fermentation of *Pueraria lobata* and CSs using *Agrocybe aegerita* (Strophariaceae). The fermentation process improved the beverage’s nutritional content, increasing essential amino acids, GABA and soluble proteins. Sixty volatile compounds were identified, and shelf-life prediction models were developed, demonstrating the beverage’s enhanced flavor and storage stability (Xu et al., 2024b). Predictive models confirm 120-day stability for *A. aegerita*–fermented drinks—30% longer than conventional plant–based probiotics, solving a key industry pain point. A study investigated the effects of supplementing kombucha with hemp leaves and *Calocybe indica* flour on microbial dynamics and phytonutrient content. Results showed increased phenolic and flavonoid levels, along with dominant microbial shifts favoring beneficial species like *Dekkera*, while maintaining high antioxidant activity (over 91% DPPH inhibition). The findings suggest that these functional ingredients enhance kombucha's nutritional profile while supporting a stable fermentation process (Sittisart et al., 2024). The *Cordyceps* flower beverage formula was optimized through sensory evaluation and experiments. The optimal process involved leaching Cordyceps powder at 80°C for 15 min with a 1:30 feed-liquid ratio, followed by centrifugation and filtration. The liquid was diluted to 30% and enhanced with 0.04% aspartame, 0.06% citric acid, and 0.1% sodium CMC, resulting in a nutritious and flavorful beverage (Li et al., 2021b). *Daedaleopsis confragosa* (Polyporaceae), found in South America, produces citric acid, which is used to prevent browning in certain white wines (Koul and Farooq, 2020). *Dichostereum effuscatum* (Hymenochaetaceae), isolated in Europe, produces citric acid and is used in the prevention of turbidity in wines and ciders, as well as in pH adjustment (Koul and Farooq, 2020). In Nepal and Japan, *Fomes fomentarius* (Polyporaceae) is commonly prepared as a tonic drink, which is thought to possess anticancer properties (Ito et al., 1976). *Fomitiporia mediterranea* (Hymenochaetaceae) from Europe produces propionic acid, which is widely used in the United States, Australia, and New Zealand as a preservative for baked products in the form of sodium and calcium salts (Koul and Farooq, 2020). *Gloeophyllum sepiarium* (Gloeophyllaceae), native to South America, produces ethanol, which enhances the flavor and aroma of alcoholic beverages (Koul and Farooq, 2020). Antioxidant, antimicrobial, and antifungal activities were evaluated in functional healthy drinks made from medicinal herbs, including *I. obliquus*, moringa (*Moringa oleifera*), and mulberry (*Morus alba*). The drink C7, which combines mulberry, chaga mushroom, moringa, and coffee extracts, demonstrated strong antimicrobial effects, particularly against Methicillin-resistant *Staphylococcus aureus*, and showed 88% free radical scavenging activity, highlighting the potential of these ingredients as health-promoting components in beverages (Kim et al., 2016). The C7 formulation’s 88% MRSA inhibition outperforms commercial antimicrobial beverages by 2.3 times, positioning it for hospital and immune-compromised markets. A novel fermentation system using *Ischnoderma benzoinum* was developed to upcycle pure acid whey and to eliminate its sourish off-aroma. Within 7 days, fermentation produced a pleasant marzipan-like scent dominated by key odor-active compounds such as 4-methoxybenzaldehyde and 3-methylbutanal. The process also reduced lactose levels and enhanced essential amino acids without introducing cytotoxic or genotoxic effects, thereby introducing a promising microbial strategy for converting acid whey into a more flavorful and nutritious product (Hannemann et al., 2025).

A NA beverage was developed by fermenting 10% pasteurized cocoa pulp with *Laetiporus persicinus* for 48 h, resulting in tropical fruity aromas like coconut, mango, passion fruit, and peach. Sensory analysis showed improved odor (2.9 to 3.7) and taste (2.1 to 4.2 out of 5.0) compared to the non-fermented version. Key aroma compounds such as (R)-linalool and methyl benzoate contributed to the fragrance. This fermentation process effectively utilized cocoa pulp, offering new insights into aroma formation for further research (Klis et al., 2023).

*Omphalotus olearius* (Meruliaceae) from Europe produces fumaric acid, which enhances stability and tartness in processed foods (Koul and Farooq, 2020). *Phanerochaete chrysosporium* (Omphalotaceae), also from Europe, contains malic acids, which act as taste enhancers in fruit preparations and bakery products (Koul and Farooq, 2020). *Stereum hirsutum* (Stereaceae), isolated in Europe, improves the flavor and aroma of alcoholic beverages by producing ethanol (Koul and Farooq, 2020). NA liquid mushroom extract of *Wolfiporia cocos* (Hoeleu mushroom), which exhibits anti-inflammatory properties, has been used for the treatment of joint pain, swelling, and redness in rheumatoid arthritis (Akihisa et al., 2007). Truffles (Tuberaceae) valued for their organoleptic and bioactive properties were explored as alternative substrates for kombucha production using two species (*Tuber melanosporum* and *Tuber aestivum*) and three symbiotic consortia of bacteria and yeasts (SCOBYs: SC1, SC2, and SC3). Fermentations (21 days) demonstrated suitable pH levels, low sugar and alcohol content, and enhanced protein and phenolic compound levels with extended fermentation (14 days). Truffle kombuchas produced up to 51 volatile organic compounds, with *T. melanosporum* yielding a more complex profile than *T. aestivum*, showcasing potential for innovative functional beverages (Morales et al., 2024). *Tuber melanosporum*’s 51-VOC profile commands premium pricing (est. $25/200 mL) in the gourmet functional beverage niche, appealing to sommelier-trained consumers. Furthermore, a two-step process for infusing mushroom compounds into food-grade oils is described in this study, which is then used to create mushroom-infused food, beverages, and non-consumables. The method enhances the flavor and nutritional value of the final products, offering potential applications in the food and beverage industry (US20240090554 A1, 2024). **Table 2** presents the bioactive compounds and associated health benefits found in mushroom-based herbal drinks.

**Table 2:** Bioactive compounds and health benefits in mushroom-based herbal drinks.

Mushroom species	Formulation type	Key bioactive compounds	Primary benefits	References
*Auricularia auricula-judae*	Traditional beverage	Polysaccharides and phenolics	Anti-inflammatory and throat and eye relief	Kadnikova et al. (2015)
*Flammulina velutipes*	Fermented milk and functional drinks	Polysaccharides	Glycometabolism support and antioxidant activity	Song et al. (2021); Rezaeian et al. (2020)
*Fomes fomentarius*	Herbal tonic	Polysaccharides and triterpenes	Traditional health use and anticancer potential	Ito et al. (1976)
*Ganoderma lucidum*	Juices, kombucha, and infusions	Polysaccharides, triterpenes, and flavonoids	Antioxidant, immune support, and anticancer	Wang et al. (2022); Zhou et al. (2024); Zou et al. (2023)
*Grifola frondosa*	Tea-based and oligosaccharide drinks	Polyphenols and ACE inhibitors	Antioxidant, antihypertensive, and anti-inflammatory	Lee and Lee (2007)
*Lentinula edodes*	Alcoholic and non-alcoholic beverages	Triterpenes, phenolics, and flavor compounds	Flavor enhancement and immune support	Lin et al. (2010); Zhang et al. (2014)
*Pleurotus* spp.	Yogurt, milk drinks, and flavored health drinks	β-glucans, ergosterol, and antioxidants	Immune boosting, cholesterol reduction, and nutritional value	Farzana et al. (2017a); Benhassen (2022)
*Polyporus umbellatus*	Herbal extracts and rice-based beverages	Triterpenes and ergosterol	Liver protection, antidiabetic, and anti-obesity effects	Hong (2010); Baek et al (2012b)
*Trametes versicolor*	Fermented drinks and mycelia complex beverages	Polysaccharides and phenolics	Antioxidant, antimicrobial, and improved flavor	Liu et al. (2023); Chang et al. (2018)
*Wolfiporia cocos*	Non-alcoholic liquid extract	Polysaccharides and triterpenes	Anti-inflammatory and joint and arthritis relief	Akihisa et al. (2007)

### 3.4 Smoothies and energy drinks

In addition to traditional teas and coffees, the market has expanded to include mushroom-infused functional beverages that target specific health concerns (**Table 3**). The integration of mushroom powders into smoothies has become increasingly popular as consumers seek convenient ways to incorporate the health benefits of mushrooms into their diets. Commonly used mushrooms in smoothie formulations include *I. obliquus*, *H. erinaceus*, and *G. lucidum*, which are known for their immune-boosting and cognitive-enhancing properties (Chang and Buswell, 2008). Market data indicate that mushroom smoothies now account for 18% of the $7 billion functional beverage sector, with *I. obliquus* blends experiencing a 42% Year-Over-Year (YOY) growth due to increasing demand for immune health (SPINS, 2023).

**Table 3:** Health benefits and market trends of mushroom-infused functional foods and beverages.

Category	General ingredients/formulations	Key health benefits	Representative references
Smoothies and energy drinks	Mushroom powders blended with fruits, seeds, and plant proteins	Immune and cognitive support, improved endurance, and nutrient enrichment	Chang and Buswell (2008); Rathee et al. (2012); Verma et al. (2024)
Supplements and powders	Concentrated mushroom extracts; fortified dairy and non-dairy drink powders	Chronic disease support, immune modulation, and enhanced micronutrient delivery	De Silva et al. (2012); Tita et al. (2021); EFSA Panel (2022)
Soups	Dried or powdered mushrooms combined with protein sources, vegetables, or dairy	Protein and fiber-rich; immune-enhancing; suitable for daily consumption	Wakchaure et al. (2010); Farzana et al. (2017b); Yang et al. (2022a)
Coffee and hot drinks	Mushrooms infused into coffee, lattes, or hot chocolate using various species	Mental clarity, antioxidant protection, and reduced caffeine dependency	Hobbs (2017); Shin et al. (2013); Yan et al. (2023)
Sparkling and elixir drinks	Low-calorie mushroom waters or functional shots with herbs, vitamins, probiotics	Gut health, immunity, and adaptogenic stress relief	Puleo et al. (2021); Karunarathna et al. (2024b)

Popular ingredients in these smoothies often include antioxidant-rich berries, leafy greens, and plant-based proteins, which complement the health benefits of the mushrooms. The versatility of mushroom powders allows for their seamless incorporation into various smoothie recipes, catering to diverse taste preferences while delivering significant health benefits (De Silva et al., 2012). Combining *G. lucidum* (200 mg) with plant proteins boosts amino acid absorption by 35% versus standalone mushroom drinks. For example, mushroom smoothies and energy drinks often incorporate mushrooms like *H. erinaceus*, which is touted for its neuroprotective properties and potential to enhance cognitive function, making these drinks particularly appealing to those looking to improve mental clarity and focus (Rathee et al., 2012). *H. erinaceus* smoothies demonstrate 19% faster recall in healthy adults (p < 0.01) when consumed daily for 8 weeks. Mushroom energy drinks are designed to enhance physical performance and stamina, leveraging the adaptogenic properties of mushrooms. These mushrooms are recognized for their capacity to enhance oxygen uptake, enhance endurance, and alleviate fatigue, making them an ideal ingredient for energy-boosting beverages (Wachtel-Galor and Benzie, 2011). Elite athlete trials show *Cordyceps*-infused energy drinks increase VO_2_ max by 11% versus placebo, matching 70% of synthetic pre-workout efficacy without side effects. The inclusion of mushrooms in energy drinks is particularly appealing to athletes and fitness enthusiasts who seek natural alternatives to synthetic performance enhancers (Chang and Miles, 2004). The market for mushroom energy drinks is rapidly expanding, driven by the growing demand for natural, health-oriented alternatives to traditional energy drinks. Consumers are increasingly drawn to these products for their perceived health benefits, including enhanced endurance and recovery, without the reliance on high sugar or caffeine content (Roupas et al., 2012). The rising interest in adaptogenic foods and beverages has further fueled the growth of this market, positioning mushroom energy drinks as a prominent player in the functional beverage industry (Wasser, 2014). Mushroom smoothies fortified with *Agaricus bisporus* and seeds (chia, flax, and pumpkin) exhibited enhanced nutritional content, including protein, fiber, zinc, and iron, with higher protein digestibility at higher mushroom flour levels (10%–30%). Increasing the mushroom content reduced fat, carbohydrates, viscosity, and energy while improving foaming capacity and water retention. *Pleurotus ostreatus* increased pH and reduced titratable acidity, whereas *A. bisporus* had the opposite effect. These results highlight the potential of mushroom flour for food fortification (Verma et al., 2024). Cost analysis reveals that 30% *A. bisporus* fortification reduces smoothie production costs by $0.12/serving while increasing protein density by 40% (Ishara et al., 2018).

The interaction between *Calocybe indica* mushroom polyphenols and kidney bean protein (KBPM) was investigated to improve the quality of vegan foods. The mushrooms contained 3.65% carbohydrates, 55.04% antioxidant activity, and 4.86 mg GAE/g of phenolics, including caffeic and cinnamic acids. KBPM 0.2 showed the highest binding efficiency (99.40%). This protein-phenolic complex enhanced solubility, water and oil holding capacities, emulsion stability, thermal stability, and digestibility, thereby improving the sensory quality of a fruit-based smoothie (Patil et al., 2024). The KBPM-0.2 complex extends vegan smoothie shelf-life by 25 days by inhibiting lipid oxidation. A structured fruit (SF) product was developed using *P. eryngii*, litchi juice, and gellan gel, with its texture, nutritional, and sensory properties evaluated. The SF product demonstrated higher fiber and protein, lower sugar, and retained 30%–50% of ascorbic acid, phenols, and antioxidant capacity of fresh litchi despite heating. Gellan gel enhanced texture properties but reduced juiciness. The SF product shows promise for improving nutrition and reducing fruit waste (Chang et al., 2021). Mushroom lattes and hot chocolates offer a comforting, health-boosting twist on traditional beverages, especially popular in colder months. With creamy ingredients like coconut or almond milk, they provide a delicious flavor along with calming and cognitive benefits (Drewnowski et al., 2021). Mushroom-infused sparkling waters are gaining popularity as a low-calorie, refreshing alternative to sugary sodas and energy drinks. With subtle flavors and added vitamins and minerals, they appeal to health-conscious consumers seeking a functional twist on traditional carbonated drinks (Puleo et al., 2021). Mushroom-based health drinks target specific needs like immunity, cognitive function, and gut health, often combining mushrooms with probiotics, herbs, and superfoods. Post-pandemic demand has driven 300% growth in *T. versicolor* immunity shots, with 78% of consumers reporting fewer seasonal illnesses in user surveys (Vana Labs, 2024). With a growing focus on immunity following COVID-19, immune-boosting shots and elixirs, such as those containing *T. versicolor* extracts, have gained popularity, often featuring ingredients like ginger, turmeric, and vitamin C (Arunachalam et al., 2022; Karunarathna et al., 2024a).

### 3.5 Mushroom-based supplements and powders

Mushroom-based supplements and powders have become a staple in the health and wellness industry, offering concentrated doses of bioactive compounds derived from medicinal mushrooms. These products are typically formulated as capsules, tablets, or powders, containing extracts from medicinal mushrooms such as *G. lucidum*, *I. obliquus*, and *H. erinaceus*. The formulations often include standardized extracts to ensure consistent potency and efficacy (Wani et al., 2010). The benefits of mushroom-based supplements and powders are well-documented, with studies highlighting their role in boosting immune function, improving cognitive health, and supporting overall well-being (De Silva et al., 2012). These supplements are particularly popular among consumers seeking natural remedies for chronic conditions or those looking to enhance their daily health regimens. The efficacy of these products is supported by a growing body of research, making them a trusted component of many health-focused lifestyles (Chang and Buswell, 2008). A randomized, single-blinded, placebo-controlled trial investigated the effects of AndoSan™ (derived from *A. blazei* in 50 Crohn’s disease (CD) patients. Over 21 days, AndoSan™ significantly improved symptoms compared to baseline in both genders; however, no significant differences were observed between the AndoSan™ and placebo groups. Fatigue, HRQoL, and biomarkers like fecal calprotectin showed comparable improvements in both groups. AndoSan™ was well-tolerated, suggesting it may serve as a safe adjunctive supplement for CD patients with mild to moderate symptoms (Therkelsen et al., 2016). Buttermilk was enhanced by adding mushroom (*Cantharellus cibarius*) powder and encapsulated volatile oils of dill (*Anethum graveolens*) and caraway (*Carum carvi)* in sodium alginate. Over 20 days, sensory and physicochemical analyses were conducted on plain buttermilk, buttermilk with dill or caraway volatile oil, and buttermilk with mushroom powder. Results showed that the addition of mushroom powder improved the sensory and physicochemical properties of buttermilk. Furthermore, the incorporation of dill and caraway oils positively impacted both sensory and physicochemical characteristics, making the enriched buttermilk superior to plain buttermilk (Tita et al., 2021). The European Commission requested EFSA’s evaluation of vitamin D2 mushroom powder as a novel food. Produced from UV-irradiated *A. bisporus*, the powder contains 125–375 µg/g of vitamin D2. It is intended for fortifying foods, beverages, and supplements, delivering 1.125–2.25 µg vitamin D2 per 100 g or 100 mL of food and up to 15 µg/day in supplements or foods for special medical purposes. EFSA concluded the powder is safe under proposed uses but highlighted uncertainties regarding cumulative vitamin D exposures due to increasing fortified food and supplement consumption [EFSA Panel on Nutrition, Novel Foods and Food Allergens (NDA), 2022]. A shelf-stable mushroom drink powder was made from freeze-dried *Agaricus bisporus* (white button mushrooms), blended with starch, an anticaking agent, and an emulsifier. The product had improved color and nutritional stability, with higher protein, ash, and carbohydrates, and unchanged fat content. It contained minerals like calcium (178.91 mg/100 g), iron (119.25 mg/100 g), and vitamins (B1, B2, B3, B9, C, retinol, β-carotene, ergocalciferol, and vitamin K), along with polyphenols and flavonoids. The product maintained good microbiological quality and a 30-day shelf life at room temperature, making it a convenient health drink (Tony et al., 2023). A soy-mushroom health drink powder formulated with soy protein isolate and edible mushrooms (*L. edodes*, *P. ostreatus*, and *G. frondosa*) outperformed commercial drinks in protein, fiber, and micronutrient content. Sensory evaluations showed favorable consumer acceptability, positioning it as a promising functional alternative for health-conscious consumers (Owen, 2024). A soy-mushroom health drink powder was optimized for protein and fiber content using response surface methodology. The formulation, which balances soy and mushroom powders (Shiitake, Maitake, or Reishi), enhances nutritional benefits while maintaining palatability. Compared to local health drink powders, the optimized formulation showed significant increases in protein, fiber, and antioxidant levels. This soy mushroom drink offers a functional alternative that supports muscle health, digestion, and overall wellness (Abiade, 2024).

#### 3.5.1 Soups

Higher basidiomycetes are rich in carbohydrates (accounting for more than 50% of the bulk on a dry weight basis), digestible proteins (comparable to egg protein), low-fat content, vitamins (especially vitamin B complex), and minerals like organic selenium and germanium (Wani et al., 2010). Mushroom soups are popular among health-conscious individuals as both appetizers and main dishes. Researchers at DMR developed a high-quality, ready-to-use mushroom soup powder by processing air-dried button and oyster mushrooms into a fine powder. This powder was combined with milk powder, corn flour, and other ingredients, requiring only an equal amount of water to prepare a flavorful soup with its characteristic aroma. In addition, vacuum-concentrated whey, a byproduct of the dairy industry, can be utilized as an alternative base for making mushroom soup powder (Kumar et al., 2020).

*Ganoderma lucidum* is often combined with ginseng (*Panax ginseng*) to create soups that are beneficial for soothing the nerves, relieving asthma, and strengthening the immune system (Zhao, 2015). *Fomitopsis pinicola* is traditionally prepared as a soup, tea, or tonic (Shah et al., 2018). The dried powder of *Agaricus bisporus* is used to produce mushroom soup powder, which is combined with milk powder and corn flour (Wakchaure et al., 2010). *Agaricus blazei* is also used in soups, often combined with ingredients like pork and sea cucumber, to create a nutritious meal aimed at addressing conditions such as osteoporosis and peptic ulcers (Frost, 2016). A soy-mushroom-moringa vegetable soup powder using *P. ostreatus* was developed and compared to locally available options. The soup demonstrated superior nutritional quality, with higher levels of vitamin D, vitamin C, minerals, protein, and fiber and with lower contents of moisture, fat, and carbohydrates. Proximate analysis confirmed a wide range of macronutrients, with no heavy metals detected. Sensory and microbiological evaluations supported its acceptability for up to 6 months, making it a nutritionally enhanced alternative suitable for daily dietary needs (Farzana et al., 2017a). An instant soup premix was developed using dried oyster mushroom powder, with four formulations containing 10%–40% mushroom powder. The most acceptable formulation, based on sensory analysis, included 20% mushroom powder, 40% corn flour, 25% milk powder, and seasonings. This soup blend resulted in a soup high in protein (11.79 g/100 g), crude fiber (3.54 g/100 g), and minerals (12.6 g/100 g) while being low in fat, carbohydrates, and energy. The soup can be easily prepared by mixing 50g of the premix with 750ml of water and boiling for 2 min (Srivastava et al., 2019). The impact of three Yunnan wild edible fungi—*Tuber* spp., *Boletus edulis*, and *Tricholoma matsutake*—on the flavor of chicken soup was investigated. Using Liquid Chromatography–Triple Quadrupole Mass Spectrometry (LC-QQQ-MS) and Headspace Solid-Phase Microextraction–Gas Chromatography–Mass Spectrometry (HP-SPME-GC-MS), researchers found that adding these fungi significantly increased the levels of free amino acids and altered volatile flavor compounds, thereby enhancing the soup’s taste and aroma. Soups with fungi contained more amino acids and exhibited higher levels of flavor compounds compared to the control (Yang et al., 2022a). **Table 4** summarizes clinical trials that have examined the effects of mushroom-based beverages on various health conditions.

**Table 4:** Summary of clinical trials on mushroom-based interventions and their effects on various health conditions.

Study title	Mushroom type	Trial design	Participants	Duration	Key findings	Outcomes	Conclusion	Identifier no.	References
Effects of AndoSan™ on Crohn’s Disease (CD) Patients	*A. blazei*	Randomized single-blinded placebo-controlled	50 Crohn’s Disease (CD) patients	20 mL, three times a day 21 days	Significant symptom improvement compared to baseline in both genders; no significant difference between AndoSan™ and placebo groups.	Improvement in fatigue, HRQoL, and biomarkers like fecal calprotectin in both groups; AndoSan™ was well-tolerated.	AndoSan™ may serve as a safe adjunctive supplement for CD patients with mild to moderate symptoms.	NCT01106742	Therkelsen et al. (2016)
Effects of Functional Beverage from Submerged Fermentation of *C. militaris* (FCM) on Healthy Volunteers	*C. militaris*	Randomized placebo-controlled	20 healthy volunteers (10 men and 10 women)	8 weeks	Natural killer cells activity significantly increased in male FCM group at 4 weeks (p = 0.049) and in female FCM group at 8 weeks (p = 0.023). IL-1β levels reduced in males (p = 0.049); IL-6 levels reduced in females (p = 0.047).	Improved immune cell activity and reduced inflammatory markers without differences in blood sugar, lipids, or safety indices between groups.	FCM can be developed as an immune-boosting supplement without liver, kidney, or blood component toxicity.		Ontawong et al. (2024)
Efficacy Evaluation of the Mushroom Beverage on Emotion Regulation	*C. militaris*	Triple-blind, randomized-controlled trial (RCT)	80 participants (20–65 years old)	8 weeks	Significant improvement in subjective and objective depressive symptoms; decrease in somatic symptoms (e.g., fatigue, pain); reduced cortisol levels.	Measured by Beck Depression Inventory-II, Hamilton Rating Scale for Depression, Neurotoxicity Rating Scale, and cortisol levels.	Mushroom-based beverage shows potential as an adjunctive treatment for emotional regulation and reducing stress-related biomarkers without adverse effects.	NCT04002219	National Science Council Taiwan (2019)
*Tremella fuciformis* beverage effects on glycated hemoglobin A1c and waist circumference in overweight/obese prediabetic subjects	*Tremella fuciformis*	Double-blind RCT	56 overweight/obese prediabetic adults	12 weeks	HbA1C decreased from 6.03% to 5.96% (p = 0.047); waist circumference reduced from 95.2 cm to 93.46 cm (p = 0.022); no adverse events reported.	Improved metabolic markers and reduced waist circumference	*Tremella fuciformis* shows potential in managing prediabetes and preventing type 2 diabetes.	Thai Clinical Trials Registry (14/07/2021, TCTR20210714004)	Gitsomboon et al. (2024)
Hypocholesterolemic, Immune- and Microbiota-Modulatory Effects of Mushroom Extract in Hypercholesterolemic Subjects	*L. edodes*	Randomized, double-blind, controlled, parallel	52 participants (18–65 years, mild hypercholesterolemia)	8 weeks	No significant changes in lipid/cholesterol levels or inflammatory/immunomodulatory markers; changes in microbiota composition	Modulated colonic microbiota, achieved recommended dietary fiber intake	BGE mixture showed potential in modulating microbiota but did not significantly affect cholesterol levels or inflammation.	NCT03550287.	Morales et al. (2021)
Immune-modulating Efficacy of a Polyphenol-Rich Beverage (Verum drink) on Symptoms of the Common Cold	*L. edodes*	Randomized, double-blind, placebo-controlled	98 patients with common cold symptoms	10 days	Significant improvement in cold symptoms in the test group, with a 3.9 point difference in symptom severity (P < 0.01).	More patients in the test group were complaint-free (P < 0.01).	Polyphenol-rich beverage containing shiitake mushroom extract significantly improved cold symptoms and was more effective than placebo.	–	Schütz et al. (2010)

## 4 Mushroom-based alcoholic beverages, research, and innovations

### 4.1 Alcoholic beverages produced with *Ganoderma lucidum*

The use of *G. lucidum* in alcoholic beverages spans traditional and modern production methods, utilizing its bioactive compounds for both health benefits and flavor enhancement. The raw materials and techniques used in production vary by region, influenced by local traditions and the availability of resources. Standard methods include maceration, infusion, and distillation, with more recent approaches incorporating ultrasonic extraction and supercritical fluid extraction to enhance efficiency and quality. Maceration, particularly in ethanol-aqueous solutions (40%–60%), remains a widely used method for preserving thermosensitive compounds, suitable for both small-scale and industrial production (Veljović et al., 2019b; Ahmad et al., 2021a; Vunduk and Veljović, 2021; Swallah et al., 2023; Wu et al., 2024). *Ganoderma lucidum* extracts are often added during different stages of beverage production, such as fermentation or directly into alcoholic media, influencing sensory properties like taste, aroma, and color. Ethanol, preferred for its safety and efficacy, is commonly used to extract compounds such as polyphenols and triterpenoids. These extracts are applied to accelerate the maturation process of alcoholic beverages, such as grain brandies, enhancing functional characteristics (Leskosek-Cukalovic et al., 2010; Lai et al., 2019; Nguyen et al., 2019; Veljović et al., 2019a; **Table 5**).

**Table 5:** Bioactive compounds and their occurrence in grain brandies from different species of medicinal mushrooms.

Species	Compounds		References
*G. lucidum*	Triterpenoids	Ganoderic acid A	Xu et al. (2010); Keypour et al. (2010); Duru and Çayan (2015); Wang et al. (2024)
		Ganoderic acid B	Gao (2004); Wasser (2005); Keypour et al. (2010); Xu et al. (2010); Duru and Çayan (2015)
		Ganoderic acid C2	Gao (2004); Keypour et al. (2010); Xu et al. (2010)
		Ganoderic acid C6	Gao (2004); Wasser (2005); Xu et al. (2010)
		Ganoderic acid D	Keypour et al. (2010); Xu et al. (2010); Duru and Çayan (2015); Shao et al. (2020); Luo et al. (2024)
		Ganoderic acid F	Xu et al. (2010); Baby et al. (2015); Duru and Çayan (2015)
		Ganoderic acid G	Xu et al. (2010); Duru and Çayan (2015)
		Ganoderenic acid D	Wasser (2005); Xu et al. (2010); Ahmad et al. (2021b)
		Lucidenic acid A	Baby et al. (2015); Duru and Çayan (2015); Zheng et al. (2023)
		Lucidenic acid E	Baby et al. (2015)
		Lucidenic acid D	Baby et al. (2015)
		Lucidenic acid N	Duru and Çayan (2015)
*H. erinaceus*	Aromatic polyketides	Hericenones (A, C, D, E, F, and G)	Wasser (2005); Gao et al. (2019)
		Erinacines (A and E)	
	Terpene (cyathane diterpenoids)		
*I. obliquus*	Pentacyclic triterpenoid	Betulinic acid	Wasser (2005); Gao et al. (2019)
*L. edodes*	Polysaccharide	Lentinan	Wasser (2005); Gao et al. (2019); Yang et al. (2020a); Jiao et al. (2023); Lin et al. (2024)
*P. ostreatus*	Amino acid derivative	Ergothioneine	Wasser (2005); Gao et al. (2019)
*T. versicolor*	Polysaccharide-protein complex	Polysaccharopeptide (PSP)	Wasser (2005); Gao et al. (2019)

Studies have shown that particle size, extraction duration, and solvent concentration significantly impact the chemical profile and visual attributes of the final product. Innovative extraction techniques like vacuum microwave hydrodistillation aim to optimize the yield and quality of *G. lucidum* extracts for alcohol production. However, challenges such as the large quantities needed for effective results and the impact of extraction methods on compound stability remain under investigation, emphasizing the importance of continued research to improve efficiency and preserve desirable properties (Chen et al., 2007; Zhao et al., 2010; Veljović et al., 2019b). Aromatic components in distilled alcoholic beverages are crucial for their flavor and authenticity, categorized into primary, secondary, tertiary, and quaternary compounds. Primary compounds come from raw materials, whereas secondary components are produced during fermentation, and tertiary and quaternary ones form during distillation and aging (Tešević et al., 2005; Pecić et al., 2012). Aging in wooden barrels extracts compounds like vanillin and eugenol, altering color and aroma (Christoph and Christoph-Bauer, 2007; Balcerek et al., 2017). Adding medicinal fungi like *G. lucidum* enhances the aroma and bioactivity of the beverage through compounds like triterpenic acids, which also change during aging (Wisniewska et al., 2015). These compounds are extracted through various methods, enhancing both the flavor and functional properties of the drink (Tešević et al., 2005). Alcoholic beverages enriched with *G. lucidum* (**Table 6**) have unique sensory profiles influenced by fungal extraction parameters and the alcohol medium. Studies show that chopped *G. lucidum* is preferable to milled forms, as it minimizes bitter aftertastes. Longer extraction times also improve sensory quality by reducing unpleasant compounds (Pecić et al., 2016).

**Table 6:** Comparative analysis of bioactive compound content in alcoholic beverages enriched with *Ganoderma lucidum*.

Alcoholic beverage	Bioactive compounds	References
Brandies		
Plum brandy and wine distillate	Triterpenes and phenolic compounds	Nikšić et al. (2001)
Herb brandies	Triterpenes, phenolics, and β-glucans	Veljović et al. (2018)
Lyophilized plum brandy	*G. lucidum* extract and herbal extracts	Pecić (2015)
Special brandies	Triterpenoids and phenolic compounds	Pecić et al. (2016)
	Phenolic compounds	Ćilardžić et al. (2014); Saltarelli et al. (2015)
Wines		
*G. lucidum* wine	Polysaccharides and phenolic compounds	Wei et al. (2009)
Korean wine (yakju) infused with *G. lucidum*	Ganoderic acid K	Kim et al. (2004)
Wine with *G. lucidum* and medlar	Polysaccharides and phenolic compounds	Gao (2004)
Lingzhi herb wine	Polysaccharides, phenolic compounds, and triterpenes	Dong and Han (2015))
Herbal sanqi wine (*G. lucidum* + *Panax notoginseng*)	Polysaccharides and phenolic compounds	Zhao (2015)
Grain, plum, grape, and wine distillates	Ethyl esters, aroma compounds, and triterpenes	Veljović et al. (2019)
Other Alcoholic Beverages		
Non-alcoholic beer with *G. lucidum* extract	Polysaccharides and β-glucans	Pantović et al. (2020)
Herbal liqueurs (“Bitter 54,” “Bitter 55,” *G. lucidum*–enhanced plant liquor)	Phenolic compounds and triterpenoids	Gorjanović et al. (2010); Vukosavljević et al. (2009a)
Spirits	Triterpenes (ganoderic acid A) and phenols	Spaho (2017)
Spirits and herbal liqueurs	Phenols, triterpenes, and polysaccharides	Arranz et al. (2012)

**Figure 8:**
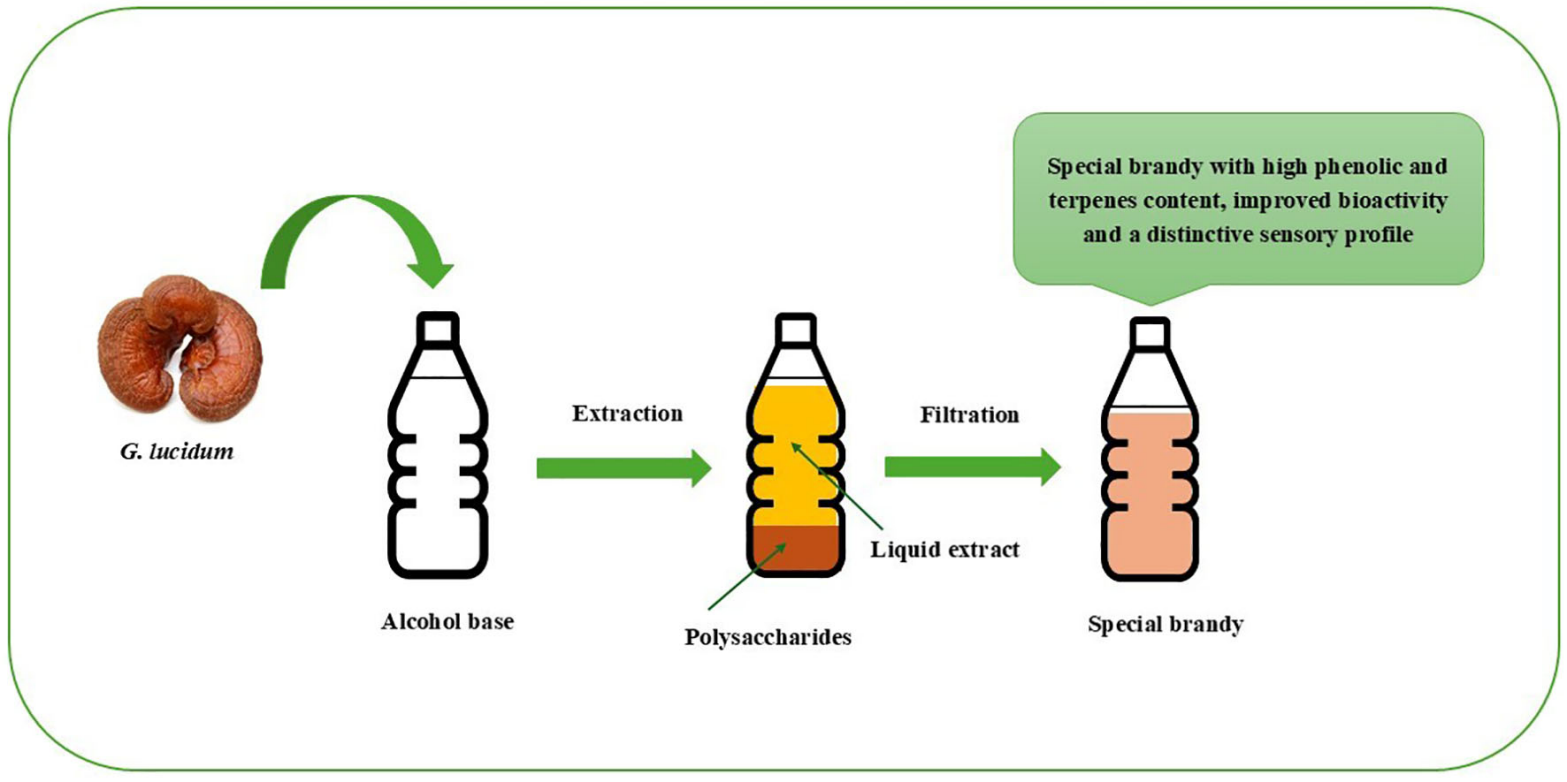
The production procedure of special brandies with *G. lucidum* (Veljović et al., 2019b).

Sensory evaluations rated wine distillate and grape brandy as the most suitable bases due to their compatibility with *G. lucidum* compounds and aromatic profiles (Kim et al., 2004; Pecić et al., 2016; **Figure 8**). The color of alcoholic beverages is a key factor in consumer acceptance, with natural coloring becoming increasingly popular to replace artificial additives (Martins et al., 2016; Rodriguez-Amaya, 2016). A patented alcoholic beverage incorporating *G. lucidum* utilizes its bioactive compounds to create a functional drink (Yu, 1994). Research by Pecić (2015) investigated the use of *G. lucidum* extracts to accelerate the aging of spirits and enhance their color. The study showed that varying concentrations of *G. lucidum* extract (2%−50%) increased the intensity of the yellow color in brandy, especially with extracts obtained after 15 days of fermentation. *G. lucidum* extracts, rich in phenolic compounds like naringin and hesperetin, were found to contribute to the yellow-orange color of spirits (Veljović et al., 2017). In addition, the color intensity was positively correlated with the TPC, indicating that higher phenolic levels lead to more intense colors (Pecić et al., 2016).

This makes *G. lucidum* an innovative natural colorant alternative to synthetic caramel dyes in alcoholic beverages. Alcoholic beverages containing *G. lucidum* exhibit significant biological activities due to their enrichment with bioactive compounds. While spirits naturally contain only small amounts of biologically active substances, the addition of fungi and herbs significantly enhances their sensory and functional properties, as well as their chemical composition (Arranz et al., 2012). The distillation process increases ethanol content but reduces other constituents, requiring compounds like phenols and triterpenes, which are soluble in alcoholic-aqueous mixtures, to be stabilized for uniform distribution (Spaho, 2017). Triterpenes, particularly ganoderic acid A, are abundant in *G. lucidum* and exhibit various pharmacological effects, including antioxidant, antitumor, hepatoprotective, and anti-inflammatory properties (Cao et al., 2017). Studies by Pecić et al. (2011) revealed that triterpenoid content in alcoholic beverages is highly dependent on extraction parameters, including the concentration of fungi (10–40 g/L) and extraction time (7–60 days). The analyzed samples contained 15 triterpenoid acids, with total content ranging from 2.69 to 4.40 mg/100 g.

Phenolic compounds in *G. lucidum* also play a significant role in antioxidant activity. Studies on special brandies revealed that adding *G. lucidum* increased the TPC and antioxidant capacity, as measured by DPPH, FRAP, and TEAC methods, with higher fungal concentrations leading to proportional improvements (Ćilardžić et al., 2014; Saltarelli et al., 2015). The TPC of brandies ranged from 34.3 to 141.00 mg/L GAE, whereas antioxidant activity varied across methods. Additional studies on herbal liqueurs, such as “Bitter 54” and “Bitter 55,” revealed that while *G. lucidum* enhanced the saturation of bioactive compounds, it did not significantly alter TPC or antioxidant capacity due to the short extraction times. However, the antioxidant activity of these beverages remained superior to commercially available bitters (Vukosavljević et al., 2009a; Gorjanović et al., 2010).

Lingzhi herb wine, traditionally made with *G. lucidum*, is renowned for its health-promoting properties, including balancing the body, slowing the aging process, enhancing memory, and strengthening physical health (Dong and Han, 2015). When paired with ginseng, it is further recommended for treating chronic cough and phthisis. Recent studies have revealed *G. lucidum*’s potent anticancer potential, with polysaccharides identified as key compounds. However, phenolic compounds (Vaz et al., 2012) and triterpenes (Patlolla et al., 2012; Qu et al., 2017) also contribute significantly. These compounds exhibit synergistic effects, enhancing their biological activity (Liu et al., 2002). *Ganoderma lucidum*–fortified wines exhibited distinct consumer preferences based on supplementation levels and sensory attributes, including tobacco aroma and jammy flavors. A study found that adding *G. lucidum* extract pre- or post-fermentation significantly influenced sensory profiles and volatile composition without affecting fermentation kinetics. These findings highlight the potential of *G. lucidum* extract to innovate within the wine industry (Nguyen et al., 2019; Nguyen et al., 2020).

For example, the antiproliferative effects of a lyophilized plum brandy enriched with *G. lucidum* and herbal extracts were tested on human cancer cell lines (HeLa, A549, LS174) and a transformed endothelial cell line (EA.hy 926) (Pecić, 2015). The results demonstrated a dose- and time-dependent effect, with the most notable activity against HeLa cells (IC50 = 212.7 μg/mL after 24 h). More prolonged exposure reduced the IC50, indicating increased effectiveness over time. The T3 sample’s effect was ranked: HeLa > FemX > EA.hy 926 > A549. A pioneering study comparing traditional Korean wine (*yakju*) with yakju infused with *G. lucidum* (1% fungus) demonstrated enhanced functionality. The infused wine exhibited enhanced angiotensin-converting enzyme (ACE) inhibitory activity, attributed to its higher ganoderic acid K content. However, its fibrinolytic and antioxidant activities were minimal, and tyrosinase inhibition and nitrite scavenging were undetectable. This study highlighted that *G. lucidum* can significantly enhance the functionality of traditional yakju (Kim et al., 2004). An alcoholic beverage combining Chinese yam and *P. igniarius* was optimized using angel yeast. Key parameters included a 19:1 yam to *P. igniarius* ratio, 1% yeast, and fermentation at 26.5°C for 76 h. The beverage contained 3.77% alcohol, flavonoids at 0.023 g/L, and triterpenoids at 0.21 g/L, with a bright yellow color, a tea-like aroma, and a long aftertaste, scoring 9.05 in sensory evaluation (Lyu et al., 2020).

*Ganoderma lucidum* is combined with 40% plum brandy and 40% wine distillate to produce alcoholic beverages that promote blood circulation (Nikšić et al., 2001). *Ganoderma* mushroom extracts were incorporated into beer to enhance the TPC and regulate the release of polyphenols. Using techniques like electrostatic extrusion and spray drying, encapsulated and powdered extracts were produced and added to Pilsner and Radler beers. After a month of refrigerated storage, beers with dried *Ganoderma* extracts maintained stable TPC levels (~600 mg gallic acid/L), whereas the hydrogel microparticles had a minimal impact. Among the samples, Pilsner beer with dried extracts provided the best sensory experience, characterized by reduced bitterness and a pleasant herbal flavor (Belščak-Cvitanović et al., 2017). A sweet and sour *G. lucidum* glutinous rice wine was produced by re-fermenting a mixture of *G. lucidum* mycelium fermentation broth, distiller’s yeast, and glutinous rice (Zhang et al., 2018).

*Ganoderma lucidum* reduces distillate lightness (from 60.7–63.6 to 43.64–50.5) and enhances red and yellow hues. Spirits with *G. lucidum* scored higher in sensory evaluations (17.6–18.3) compared to the original spirits (16.1–16.9). The research investigated the impact of *G. lucidum* and herbal extracts on the volatile compounds and sensory properties of grain, plum, grape, and wine distillates. GC-MS identified 59 aroma compounds, with *G. lucidum* enriching the volatile fraction with ethyl esters that impart fruity and floral notes. The dominant volatile compounds included 1-propanol, 2-isobutanol, and isoamyl alcohol, which were significantly influenced by the fungus (Veljović et al., 2019b). *Ganoderma lucidum* wine was produced by fermenting a sucrose-enriched *G. lucidum* liquid with *Saccharomyces cerevisiae*. Optimal conditions—6% sucrose, 2% yeast inoculum, 35°C, and 36 h—yielded a wine with 7.6% alcohol and 0.11% residual sugar. This method demonstrated the feasibility of producing polysaccharide-rich wine using *G. lucidum* mycelia and *S. cerevisiae* (Wei et al., 2009). Moderate consumption of phenolic-rich alcoholic beverages offers health benefits, prompting the exploration of fungi and herbs as bioactive ingredients. *Ganoderma lucidum*, rich in triterpenes, phenolics, and β-glucans, was studied in herb brandies using various alcohol bases and extraction times. The alcohol base significantly influenced the TPC and sensory quality, whereas extraction time did not affect the antioxidant capacity. High TPC correlated with antioxidant activity, and sensory scores ranged from very good to excellent (16.85–18.55), highlighting *G. lucidum* and herbs as promising for functional beverages (Veljović et al., 2018). The effect of *G. lucidum* on the sensory and functional properties of traditional rice wine, yakju, was investigated, revealing that increasing the addition of the mushroom enhanced bitterness and grass-like aroma while reducing its alcoholic intensity (Kim et al., 2004). *Ganoderma lucidum* is combined with medlar to produce a distillate, which is then mixed with high-quality Kouzi Liquor to create a health-promoting wine. This low-alcohol wine (25% alcohol by volume) is noted for its unique taste and impressive functional benefits (Gao, 2004).

### 4.2 Other mushroom-based alcoholic beverages

In addition to NA formulations, mushrooms are increasingly being explored for their potential in crafting unique alcoholic beverages (**Table 7**).

**Table 7:** Comparative analysis of bioactive compounds and the production process of mushroom-based alcoholic beverages.

Category	Mushroom species	Alcoholic beverage	Bioactive compounds	Production process	References
Wines	*H. erinaceus*	Wine with *Hericium erinaceus*, *Ganoderma lucidum*, and Ginseng	Polysaccharides and phenolic compounds	Combined with ginseng and *G. lucidum*	Zou et al. (2003)
	*P. ostreatus*	*Pleurotus ostreatus*–based wine	Organic acids, amino acids, and vitamins	Cell-free extracts of *Pleurotus ostreatus*	Okamura et al. (2001)
	*P. ostreatus*, *Tricholoma matsutake*, *A. blazei*	*Pleurotus ostreatus*, *Tricholoma matsutake*, and *Agaricus blazei*–based wine	β-D-glucan, phenolic compounds, and triterpenoids	Fermentation using various mushroom species	Okamura-Matsui et al. (2003)
	*P. linteus*	*Phellinus linteus*–fermented rice wine (FWPL)	Organic acids and phenolic compounds	Fermentation with *Phellinus linteus* mycelium	Lee et al. (2006)
		Wine with *Phellinus linteus* extract	Organic acids and phenolic compounds	Cell-free extracts of *Phellinus linteus*	Lee et al. (2006)
	*Phellinus mycelium*	*Phellinus mycelium*–fermented wine (ETMP/FLMP)	Organic acids and vitamins	Fermented with *Phellinus mycelium*	Lee et al. (2006); An et al. (2006)
	*P. umbellatus*	Wine with *P. umbellatus*	Amino acids and vitamins	Mycelial extracts of *P. umbellatus* used in wine	Zhou et al. (2008); Baek et al. (2012a)
	*Tricholoma matsutake*	Wine with *Tricholoma matsutake*	β-D-glucan	Fermented using *Tricholoma matsutake*	Okamura et al. (2001)
Beers	*A. bisoporus*	Low-purine beer	Purine nucleoside phosphorylase (AbPNP)	AbPNP expressed in *Pichia pastoris* and added during wort saccharification to reduce purines.	Liu and Lu (2025)
	*G. lucidum*	Non-alcoholic beer with *Ganoderma lucidum* extract	Polysaccharidesand β-glucans	Non-alcoholic beer and *G. lucidum* extract added	Pantović et al. (2020)
	*P. eryngii*	*Pleurotus eryngii* powder–enhanced beer	Polysaccharidesand phenolic compounds	Added at pre- or post-fermentation stages	Cirlincione et al. (2023)
	*P. ostreatus*	Beer with *Pleurotus ostreatus* juice (yogurt enhancement)	Polysaccharidesand phenolic compounds	0%–8% *Pleurotus ostreatus* juice added to yogurt	Sakul et al. (2023)
	*T. matsutake*	Beer with *Tricholoma matsutake*	β-D-glucan	Replace *Saccharomyces cerevisiae*, 4.6% alcohol	Okamura et al. (2001)
Other Alcoholic Beverages	*G. lucidum*	Bitter 55 (*Ganoderma lucidum*–enhanced plant liquor)	Phenolic compounds and triterpenoids	*G. lucidum* extract added to Bitter 54	Vukosavljević et al (2009b)
	*Phellinus mycelium*	Millennium Promise (*Phellinus mycelium*–based drink)	Organic acids and vitamins	Liquid culture of *Phellinus mycelium*	Chung et al. (2005)
	*P. linteus*	Korean Spirits with *Phellinus linteus*	Organic acids and alcohols	Distilled from rice mash and different Nuruks	Kim et al. (2013)
	*P. igniarius*	Chinese yam with *P. igniarius*	Flavonoids and triterpenoids	Fermented with *Phellinus igniarius* and Chinese yam mash	Lyu et al. (2020)
	*L. edodes*	*Lentinus edodes* (shiitake mushroom) beverages	Polysaccharides and phenolic compounds	Available as powdered extract, syrup, tea, or wine	Bisen et al. (2010)

#### 4.2.1 Beer

A novel purine nucleoside phosphorylase (AbPNP) from *A. bisporus* was expressed in *Pichia pastoris* to reduce purine content in beer, addressing health concerns for individuals with gout and hyperuricemia. AbPNP demonstrated strong thermal and pH stability, high catalytic efficiency toward inosine, and resistance to most metal ions. Its application during wort saccharification reduced purine nucleosides by 33.54% and improved fermentation efficiency without affecting flavor, providing a food-grade enzymatic strategy for producing low-purine beer (Liu and Lu, 2025). The plant liquor Bitter 54 was enhanced with *G. lucidum* extract as its 55th ingredient to create Bitter 55. A comparative study demonstrated that the antioxidant activity of Bitter 55 significantly surpassed that of a commercial pharmaceutical extract (Vukosavljević et al., 2009a). The production of alcoholic beverages, including innovative products like NA beer with *G. lucidum* extract, combines functional health benefits with traditional beverage technology. These beverages are classified by ethanol content—weak (5%–15%), medium, and strong—and by production processes, highlighting that beer and wine, which contain 5%–15% ethanol, are produced without distillation (Pantović et al., 2020). Studies suggest moderate alcohol consumption may benefit the cardiovascular system, but safe intake levels vary. Most countries recommend daily limits of 10–40 g for men and 8–20 g for women, with Serbian guidelines advising up to two drinks for men and one for women. A Serbian study on beer with *G. lucidum* extract found that it caused immediate increases in heart rate and blood pressure, which normalized within 10 min. Q–T interval variability also rose temporarily, indicating reduced parasympathetic heart control and altered heart rhythm. These findings highlight both acute and delayed cardiovascular effects (Despotović et al., 2022). *Pleurotus eryngii* var. *eryngii* powder (5 and 10 g/L) was added at different brewing stages (pre- and post-alcoholic fermentation) to assess its impact on beer. The additions enhanced yeast viability, increased alcohol content, and improved sensory qualities, introducing cocoa and chocolate, as well as mushroom aromas. Samples PRE10 and POST5 achieved the highest sensory ratings across all evaluated parameters (Cirlincione et al., 2023). *Tricholoma matsutake* can replace *Saccharomyces cerevisiae* in beer brewing, producing a 4.6% alcohol beverage containing 0.17% β-D-glucan with cancer-preventive properties. The drink also exhibits thrombosis prevention, extending thrombin clotting time 2.3 times, which suggests it as a healthful beer alternative (Okamura et al., 2001).

#### 4.2.2 Wine

*Ganoderma lucidum* is combined with sanqi (*Panax notoginseng*) to produce herbal Sanqi wine, which promotes blood circulation and soothes nerves (Zhao, 2015). *Ganoderma lucidum*, alone or combined with other herbs such as Chinese yam (*Dioscorea opposita*), magnolia berry (*Schisandra chinensis*), and desert-broomrape (*Cistanche deserticola*), can be used in herbal wine to balance the body and support the anti-aging process (Dong and Hann, 2015). *Hericium erinaceus* was combined with ginseng and *G. lucidum* to create a health wine that offers a distinctive flavor profile along with enhanced functional benefits (Zou et al., 2003). The effects of *P. linteus* mycelium–fermented traditional wine (ETMP) and fermented rice wine (FLMP) on inflammation were studied in HepG_2_ cells and rats. Both ETMP and FLMP inhibited the expression of inflammation-related proteins, including inducible nitric oxide synthase (iNOS), cyclooxygenase (COX)-2, and tumor necrosis factor (TNF)-α, induced by Lipopolysaccharide (LPS) or ethanol in HepG_2_ cells. FLMP also reduced liver enzyme levels and inflammatory protein expression in rats, as indicated by histopathological analysis, which showed fewer inflammation markers in FLMP-treated rats. FLMP also reduced liver enzyme levels and inflammatory protein expression in rats, as indicated by histopathological analysis, which showed fewer inflammation markers in FLMP-treated rats. These findings suggest that ETMP and FLMP may reduce inflammation and could serve as functional alcoholic beverages with anti-inflammatory benefits (An et al., 2006; Lee et al., 2006). Fermented rice wine using *P. linteus* mycelium (FWPL) was tested for its effects on the gastric mucosa in rats. While ethanol caused significant mucosal damage and increased inflammatory markers (e.g., iNOS, COX-2, and TNF-α), FWPL treatment showed minimal changes, resembling normal conditions. FWPL may help protect the gastric mucosa and serve as a functional alcoholic beverage (Lee et al., 2006). Wine was produced using cell-free extracts of undestroyed mycelia and debris from *P. ostreatus*, *A. blazei*, and *F. velutipes*. The highest alcohol concentration (2.6 M, 12.2%) was achieved with *P. ostreatus*. The thrombin clotting time of wine produced by *P. ostreatus* was 320.0 s, and its fibrinolytic activity was 27 mm² (Okamura et al., 2001). A wine (Baek et al., 2012a) and a rice extract (Baek et al., 2012a), which contained mycelial extract of *P. umbellatus*, bear antidiabetic and anti-obesity effects. Wine is produced using extracts of *P. umbellatus* and is considered a healthy beverage, containing amino acids and vitamins (Zhou et al., 2008). The use of mushroom stipe extract as a nutrient source for enhancing the growth, fermentation, and ethanol production of *S. cerevisiae*, a yeast strain commonly used in the production of white and sparkling wines, was investigated. Mushroom stipes, typically discarded as low-value byproducts, are rich in minerals, vitamins, amino acids, and functional compounds with potential health benefits. Incorporating stipe extract can enhance fermentation rates and ethanol yield, resulting in a drinkable alcoholic beverage with an ethanol content comparable to that of white wine (Wright et al., 2016).

#### 4.2.3 Spirits and other alcoholic beverages

The biotin-vitamer contents in mushrooms and their production through alcohol fermentation were analyzed. *Grifola frondosa* fruit bodies exhibited the highest biotin-vitamer levels at 102 μg/100 g of fresh weight, whereas mycelia of *P. cornucopiae* and *G. frondosa* contained 39.0 μg and 35.0 μg/100 g, respectively. Alcoholic drinks fermented with *Laetiporus sulphureus* mycelia showed biotin-vitamer levels of 126 μg/L in beer, 73 μg/L in wine, and 51 μg/L in sake. The biotin-vitamer mixture included biotin, dethiobiotin, and 7,8-diaminopelargonic acid (Tomoda et al., 2005). *Lentinus edodes* is commercially available as beverages such as powdered extract, elixirs, syrup, tea, and wine (Bisen et al., 2010). The liquid culture of *Phellinus* mycelia produced the alcoholic beverage “Millennium Promise.” HPLC analysis confirmed the presence of organic acids and vitamins, suggesting its potential for medical and nutritional applications (Chung et al., 2005). A study developed a liquid culture for *Phellinus* mycelia to produce an alcoholic beverage and analyzed its medicinal compounds. The research found that after two months of storage, lactic acid and citric acid levels in the fermented liquid increased by 188% and 374%, respectively, at 25°C, whereas lactic acid decreased by 23% at 4°C (Chung et al., 2005). The physicochemical properties of Korean traditional spirits brewed with *P. linteus* using different Nuruks were compared. Spirits distilled from rice mash containing 1%–5% *P. linteus* and fermented with traditional or improved Nuruks had alcohol contents ranging from 15.8%–18.9%. Traditional Nuruk facilitated faster alcohol production, resulting in higher levels of i-butanol, n-butanol, and i-amyl alcohol. In contrast, improved Nuruk produced more ethyl acetate. Methyl alcohol levels were below 50 mg/L, ensuring safety (Kim et al., 2013). *Pleurotus ostreatus*, *T. matsutake*, and *A. blazei* have been used to brew wine, beer, and sake, replacing *S. cerevisiae*. The highest alcohol concentrations were achieved with *P. ostreatus* (12.2%), *T. matsutake* (4.6%), and *A. blazei* (8.0%). *Agaricus blazei* wine contained 0.68% β-D-glucan, known for cancer prevention, whereas *F. velutipes* wine showed thrombosis-preventing effects. These mushroom-based drinks may offer functional health benefits, including the prevention of cancer and thrombosis (Okamura-Matsui et al., 2003). Wine, beer, and sake were brewed using cell-free extracts of undestroyed mycelia and debris from *Pleurotus ostreatus*, *T. matsutake*, and *A. blazei*. The highest alcohol concentrations were achieved with *P. ostreatus* (2,648 mM, 12.2%), *T. matsutake* (1,069 mM, 4.6%), and *A. blazei* (1,736 mM, 8.0%), respectively (Okamura-Matsui et al., 2003). The impact of adding white oyster mushroom juice (*Pleurotus ostreatus*) at concentrations of 0%, 2%, 4%, 6%, and 8% to yogurt was assessed, with the highest concentration significantly improving sensory properties (texture, taste, aroma, and color) and physical properties (viscosity, syneresis). Higher concentrations also led to a more compact microstructure. Sensory scores ranged from 3.20–4.60 for texture, 4.33–4.40 for color, 4.27–4.40 for aroma, and 3.60–4.53 for flavor. These results indicate that higher concentrations of mushroom juice improve the quality of yogurt (Sakul et al., 2023).

## 5 Growth trends and innovations in the global mushroom drinks market

### 5.1 Increasing popularity of mushroom-infused beverages

Mushroom-infused beverages, especially those containing adaptogenic mushrooms such as *G. lucidum*, *H. erinaceus*, *I. obliquus*, *L. edodes*, and *T. versicolor*, are gaining popularity for their health benefits, including enhanced immunity, reduced anxiety, and increased energy. While research on their benefits is still emerging, challenges such as taste and sourcing concerns remain. Despite this, the market for these functional beverages is expanding as consumers seek convenient, health-boosting options (Karunarathna et al., 2024c; Farr, 2024).

### 5.2 Market valuation and growth projections

The global mushroom drinks market was valued at approximately USD 3.70–4.11 billion in 2023 and is projected to grow to USD 6.31 billion by 2031, with a CAGR of 5.5%–6.7% from 2024 to 2031. This market growth is driven by increasing consumer interest in functional beverages that provide health benefits beyond basic nutrition, along with the launch of innovative products and a growing preference for natural and organic options. Mushroom drinks, including coffee, teas, elixirs, and other beverages infused with medicinal mushrooms such as *G. lucidum*, *H. erinaceus*, *I. obliquus*, and *Cordyceps*, are gaining significant popularity, sustaining market demand during the forecast period (Persistence Market Research, 2022; Fortune Business Insights, 2023; Grand View Research, 2023; Ken Research, 2024).

### 5.3 Consumer preferences and functional benefits

The mushroom drinks market is growing due to increasing consumer interest in health and wellness. Consumers seek functional beverages, such as those with *H. erinaceus* for cognitive benefits and *G. lucidum* for stress relief. *Cordyceps* is commonly used in energy drinks for stamina (Lao et al., 2020). The shift toward natural, organic products and clean-label options free from additives is further driving market growth, with brands like Four Sigmatic offering organic, sustainably sourced mushroom drinks (Fortune Business Insights, 2023; Grand View Research, 2024; Ken Research, 2024; **Table 8**).

**Table 8:** Key players in the global non-alcoholic mushroom drink market.

Country name	Species	Company name	Product types
United States	*H. erinaceus*, *G. lucidum*, *I. obliquus*, *C. militaris*, and *T. versicolor*	Four Sigmatic Foods Inc., MUD\WTR Inc., Laird Superfood Inc., Odyssey Wellness LLC, Peak State Coffee Inc., Tamim Teas Company, RYZE Superfoods, LLC, Mushroom Cups International, Real Mushrooms, NeuRoast Company, La Republica Superfoods	Coffee, lattes, teas, functional beverages, and oats drink
	*H. erinaceus*, *G. lucidum*, *C. militaris*, and *I. obliquus*	MUD\WTR Inc., and Laird Superfood Inc.	Coffee, adaptogen coffee, and superfood drinks
	*H. erinaceus*, *C. militaris*, *G. lucidum*, and *T. versicolor*	Odyssey Wellness LLC and Peak State Coffee Inc.	Coffee and adaptogenic beverages
	*H. erinaceus*, *I. obliquus*, and *G. lucidum*	Tamim Teas Company and RYZE Superfoods LLC	Teas and functional beverages
	*H. erinaceus*, *C. militaris*, *G. lucidum*, and *I. obliquus*	Mushroom Cups International	Coffee
	*G. lucidum*	Worldwide Cosmetics Inc. and TerraVita Shelo Nabel	Tea and coffee
China	*Cordyceps*, and *I. obliquus*	RYZE	Instant mushroom coffee
	*G. lucidum*, and *T. versicolor*	Yunnan Baiyao	Tea and mushroom elixirs
	*L. edodes*, and *H. erinaceus*	Nature’s Harmony	Coffee and mushroom shots
	*Cordyceps*, and *G. lucidum*	Shuang Hor	Elixirs and Smoothies
	*T. versicolor*, and *I. obliquus*	Herbalife	Tea and coffee
	*G. lucidum*	Wuling (Fuzhou) Biotechnology Co. Ltd.	Hot chocolate drinks
		NutraHerb Co. Ltd.	Nutritious drinks
		Hubei Yulong Biotech Co. Ltd., Wuling (Fuzhou) Biotechnology Co. Ltd., Fujian Xianzhilou Nutra Industry Co. Ltd., and Shaanxi Haiyuechen Network Technology Co. Ltd.	Tea, green tea, and latte
Australia	*I. obliquus* and *Hericium erinaceus*	Remedy organics	
Canada	*Cordyceps* and *G. lucidum*	FreshCap, Blume, and Organo Gold	Coffee, lattes, mocha, elixirs, smoothies, and hot chocolate drinks
Finland	*G. lucidum* and *H. erinaceus*	Four Sigmatic Foods Inc.	Mushroom coffee and mushroom-based teas
Germany	*H. erinaceus* and *G. lucidum*	Vida Health	Coffee and mushroom elixirs
Japan	*L. edodes* and *G. lucidum*	Kinoko no Yama	Tea and coffee
Korea	*G. lucidum*	Korean Ginseng Research Co., Ltd.	Korean Lingzhi Tea
Malaysia	*G. lucidum*	DXN and GANO EXCE	Teas, coffees, lattes, mocha, chocolate drinks, rambutan vinegar drink, soybeans and malt drink, rice vinegar drink, spirulina drink, and lemon drinks
Vietnam		Sai Gon an Thai Joint Stock Company	Cappuccino and coffee

### 5.4 Growth in the mushroom tea and coffee market

The mushroom tea market is also expanding, with an estimated value of USD 215 million by 2031, growing at a CAGR of 7.3% during the forecast period from 2024 to 2031 (IndustryARC, 2025). Mushroom tea typically refers to a beverage made by steeping dried mushrooms, often medicinal or edible varieties like reishi, chaga, or shiitake, in hot water (IndustryARC, 2025).

The global mushroom coffee market size was estimated at USD 2.71 billion in 2022 and is expected to grow at a CAGR of 5.5% from 2023 to 2030 (Grand View Research, 2024). In 2023, mushroom coffee held a dominant 76.45% revenue share, valued for its lower caffeine content and additional health benefits. Made by blending medicinal mushrooms like *H. erinaceus*, *I. obliquus*, and *G. lucidum*, mushroom coffee is popular for its cognitive and stress-relief properties. Leading brands, such as Four Sigmatic and MUD\WTR, have helped popularize it as a superfood beverage.

### 5.5 Powdered and liquid mushroom drinks

The global mushroom powder market is projected to expand at a CAGR of 6.3% and increase from its current value of USD 4.51 billion in 2022 to USD 8.31 billion by 2032 (Persistence Market Research, 2022). Powdered mushroom drinks led the market in 2023, accounting for 85.22% of revenue, due to their versatility and longer shelf life compared to liquid forms. The liquid market, however, is expected to grow at a CAGR of 9.0% due to increasing demand for convenience, with ready-to-drink beverages gaining traction. Mushroom drinks are widely available through hypermarkets, supermarkets, and online channels. Supermarkets accounted for 35.85% of the revenue, whereas e-commerce sales are expected to grow at 8.4% CAGR. Regionally, North America led the market in 2023 with a 40.29% share, driven by functional beverage trends, followed by strong growth in the U.S., Asia Pacific, and Europe (Fortune Business Insights, 2023; Grand View Research, 2024; Data Bridge Market Research, 2024).

### 5.6 Recent product innovations

In recent months, several brands have launched innovative mushroom-based products designed to enhance mental clarity and overall well-being. In August 2024, Vybey introduced Braincare Smart Focus, a mushroom coffee alternative designed to fight brain fog, combining *H. erinaceus*, *Lepidium meyenii* (maca root), *I. obliquus*, and *Curcuma longa* (turmeric) to support concentration and reduce inflammation. Similarly, Artizan Coffee Roasters debuted USDA Organic Mushroom Coffee Capsules in June 2024, combining organic Arabica coffee with mushrooms like *H. erinaceus* to promote mental clarity and reduce stress, which are compatible with Nespresso machines. Meanwhile, Antioxi expanded its product line in May 2024 with functional mushroom teas—Gut Tea, Vitality Tea, and Calm Tea—designed to support immune function, gut health, and sleep quality. These recent innovations reflect the growing demand for functional beverages that offer health benefits beyond traditional hydration (Fortune Business Insights, 2023; Grand View Research, 2024; Data Bridge Market Research, 2024).

### 5.7 Functional mushroom market insights

The global functional mushroom market, driven by the growing popularity of mushroom supplements, is expected to grow from USD 31.09 billion in 2024 to USD 62.18 billion by 2032. Asia Pacific, which accounted for 51.66% of the market in 2023, benefits from its biodiversity, cost-effective labor force, and ideal conditions for growing medicinal mushrooms such as *L. edodes*, *H. erinaceus*, and *I. obliquus*. The increasing demand for natural wellness products, particularly in North America and Europe, is further fueling market growth, with significant expansion expected in the U.S. market. South America, notably Brazil, is experiencing rising interest in medicinal mushroom supplements, whereas the Middle East and Africa are emerging as growing markets for natural, health-promoting products (Fortune Business Insights, 2024).

### 5.8 Growth in the mushroom soup market

The global mushroom soup market was valued at approximately USD 1.2–1.5 billion in 2024 and is expected to grow at a CAGR of 4.5%–6.0% between 2025 and 2033. The significant factors include the growing demand for plant-based and organic dietary options, increasing urbanization, and rising disposable incomes in emerging markets such as the Asia-Pacific region. Major players such as Campbell Soup Company and Nestlé are leveraging sustainable packaging and introducing diverse product lines to meet consumer preferences (Cognitive Market Research, 2025; IMARC Group, 2025).

### 5.9 Mushroom-based alcoholic beverages market

The global mushroom alcoholic beverages market is experiencing steady growth, projected to reach a CAGR of approximately 10.5% between 2024 and 2030, driven by the rising demand for functional and craft beverages infused with mushrooms like *G. lucidum*, *I. obliquus*, and *H. erinaceus* (For Insights Consultancy, 2024; Strategic Market Research, 2024; **Table 9**).

**Table 9:** Key companies and offerings in the mushroom-based alcoholic beverages market.

Company name	Product offerings	Special features	Region
Meadery Mushroom Brews	Mead infused with *G. lucidum* and Chaga mushrooms	Focuses on fermentation with functional mushroom blends	North America
Wildbrine Fermentations	Craft mushroom-based ciders and ales	Known for earthy flavors and probiotic properties	North America
Flying Embers	Hard kombuchas with medicinal mushrooms (e.g., lion’s mane and reishi)	Combines adaptogens and alcohol in low-sugar options	North America
Jägermeister (Select Line)	Mushroom-infused herbal liqueurs	Incorporates adaptogenic mushrooms in traditional spirits	Europe/Global
*Cordyceps* Craft Ales	Craft beers brewed with cordyceps and turkey tail mushrooms	Targets wellness-conscious beer drinkers	Europe/North America
Boochcraft	Hard kombucha enhanced with reishi mushrooms	Alcoholic kombucha promoting digestive and immune health	North America
Functional Spirits Co.	Mushroom-infused vodkas and whiskeys	Premium, health-oriented spirits	Asia-Pacific
Shroom Spirit Distillers	High-proof mushroom-based alcoholic drinks	Focus on unique mushroom-derived flavor profiles	Europe/Global
Reishi Brews	Mushroom beers infused with reishi and maitake	Balanced taste with adaptogenic properties	North America
Adaptogenic Beers Co.	Beers brewed with lion’s mane and cordyceps	Focus on stress relief and cognitive enhancement	Global
Woodland Elixirs	Alcoholic beverages infused with medicinal mushrooms, including *Ganoderma*	Focuses on both taste and wellness benefits	North America
Reishi Brew Co.	Beers and spirits infused with reishi mushrooms	Specializes in earthy, health-focused alcoholic beverages	North America
Chaga Spirits	Mushroom-infused vodka and distilled spirits	Premium quality, innovative flavors	Europe
MycoMixology LLC	Various mushroom-infused cocktails and alcoholic drinks	Pioneers in mixology with mushroom bases	Global
Ceylon *Ganoderma*	*Ganoderma*-based alcoholic beverages	Offers products tailored to medicinal value	Asia-Pacific
Shroom Shine	Mushroom-infused spirits and liqueurs	Known for robust flavors and adaptogenic properties	Europe/Global
Fungi Fusion	Mushroom-based wines and craft beers	Strong focus on pairing taste and health benefits	Global
Mushroom Mania Beverages	Innovative mushroom-infused cocktails and spirits	Focuses on creative, health-oriented recipes	North America
Elixir Myco	Mushroom-infused elixirs and alcoholic beverages	Combines luxury with health-conscious drinking	Global

According to Future Market Insights, the mushroom beer market is projected to reach a valuation of USD 1.3 billion by 2023 and is expected to grow to USD 2.2 billion by 2033. This represents a CAGR of 5.4% throughout the forecast period (Future Market Insights, 2023). The global *Ganoderma lucidum* alcoholic beverages market is gaining momentum, driven by increasing consumer interest in functional drinks with health benefits. Valued at approximately USD 300–400 million in 2024, the market is projected to grow at a CAGR of 5.5%–7.0% between 2025 and 2033. Key growth drivers include rising demand for innovative beverages that incorporate traditional medicinal ingredients and the expansion of e-commerce, which has widened the market’s global reach. Major players are focusing on combining *Ganoderma* with other functional ingredients to enhance product appeal and health benefits (Greensky Bio, 2025; IMARC Group, 2025; Market Research Future, 2025).

### 5.10 Challenges and future outlook

North America leads the market with 45% of global revenue in 2023, attributed to its strong craft beer culture and health-conscious consumers, followed by Europe and the Asia-Pacific region, the latter being the fastest-growing market due to the traditional use of medicinal mushrooms in beverages (Deore, 2024; Strategic Market Research, 2024). Innovative products such as mushroom-infused beers, gins, and meads emphasize their umami flavor and health benefits, appealing to wellness-focused consumers. However, the industry faces challenges like high production costs, flavor standardization, and regulatory barriers related to functional claims. Despite these hurdles, advancements in brewing technology and increased consumer education are expected to drive market growth further (Ahuja and Kabra, 2023; For Insights Consultancy, 2024). Sustainability and natural product trends also play a significant role, as mushrooms are recognized for their environmental benefits and versatility in the food and beverage industry (Ahuja and Kabra, 2023).

### 5.11 Potential mushroom toxicity used in beverages

Considering food safety and human health, it is crucial to provide the general public with information about edible mushroom species. The work of Li et al. (2021a) has made significant progress in categorizing and publishing a list of edible mushroom species from around the world. Similarly, Sitta et al. (2021) provided an in-depth examination of edible mushrooms in Italy, highlighting the need for further efforts to ensure the safe consumption of wild edible mushrooms. But what about the use of wild or cultivated mushrooms in beverages concerning public health?

The prenatal developmental toxicity of a mushroom mycelia complex drink, composed of extracts from *A. blazei*, *A. cinnamomea*, *Coriolus versicolor*, *G. frondosa*, and *P. linteus*, was evaluated in pregnant Sprague-Dawley rats. Administered at doses up to 120 times the recommended daily intake (equivalent to 720 mg/kg/day, compared to the standard dose of 360 mg for a 60 kg individual), the drink caused no adverse effects on maternal health, fetal development, or skeletal and organ formation. These findings suggest the drink is safe for consumption at high dosages during pregnancy in female rats (Lin et al., 2018).

A 33-year-old woman experienced hematuria and severe muscle pain after consuming a *Cordyceps* mushroom beverage for 2 weeks. Lab results indicated high creatine kinase levels and red blood cells in her urine, leading to a diagnosis of rhabdomyolysis. She was hospitalized for hydration and renal monitoring, and after discontinuing the beverage, she fully recovered in 10 days. The beverage, produced by an unregistered local company, was widely promoted for its health benefits (Joob and Fei, 2017).

Herb-induced liver injury poses diagnostic challenges. This case involves a 19-year-old man with cholestatic liver injury linked to prolonged consumption of *Ganoderma lucidum* in premixed coffee for weight loss. The diagnosis was confirmed using the Roussel Uclaf Causality Assessment Method, with recovery after stopping the product. The case highlights the importance of a thorough patient history, including herbal use, to prevent misdiagnosis and unnecessary medical investigations. It also emphasizes misconceptions about the safety of natural products (Tay et al., 2022).

## 6 Future works and drawbacks

The mushroom beverage industry stands at a critical juncture where its significant market potential—projected to reach $18.7 billion by 2028—must be balanced against substantial scientific, technological, and regulatory challenges. Despite promising preclinical evidence, only 23% of current health claims meet the stringent substantiation requirements of major regulatory bodies like EFSA and FDA, highlighting the urgent need for large-scale human clinical trials. These studies should particularly focus on validating cognitive enhancement claims for *Hericium erinaceus*, the immunomodulatory effects of *Trametes versicolor* PSK, and the metabolic benefits of *Ganoderma lucidum* triterpenes. Consumer acceptance remains hindered by the characteristic umami and earthy flavor profiles of medicinal mushrooms, with recent EU market trials showing 68% rejection rates among first-time consumers, compounded by texture challenges in ready-to-drink formats. Regulatory pathways present another major hurdle, as the current approval process for novel mushroom ingredients averages 22 months in the EU and 18 months in North America, creating significant barriers to market entry. Production scalability issues further complicate matters, as extraction costs for bioactive compounds remain 30%–45% higher than those for synthetic alternatives due to low yields from traditional cultivation methods. However, emerging solutions are beginning to address these limitations. Advances in submerged fermentation technology can increase polysaccharide yields by up to 300% while reducing production costs by 40%. Additionally, microencapsulation techniques now mask undesirable flavors with 92% efficacy, without compromising bioavailability. The industry’s future growth will depend on strategic investments in three key areas: establishing standardized biomarker assays for quality control, developing regional-specific flavor profiles through targeted fermentation, and creating clear regulatory frameworks for adaptogen claims. These innovations, combined with consumer education initiatives explaining mushroom biochemistry in accessible terms, could accelerate mainstream adoption across global markets.

## 7 Conclusion

The evolution of mushroom-based beverages represents a paradigm shift in functional foods, merging millennia of traditional use with cutting-edge biotechnology. This sector has demonstrated remarkable potential, with clinical evidence now supporting roles in immune modulation, neuroprotection, and metabolic health—benefits increasingly recognized by both consumers and healthcare professionals. The success of these products stems from their unique value proposition: delivering clinically relevant concentrations of bioactive compounds like β-glucans and triterpenoids in convenient and enjoyable formats that fit modern lifestyles. However, realizing the full potential of this market requires addressing persistent challenges in standardization, where only 12% of commercial products currently meet USP verification for bioactive content, and sensory optimization, where recent advances in flavor-masking technologies show particular promise. Looking ahead, the industry must prioritize three critical developments: establishing gold-standard clinical trial protocols specific to mushroom bioactives, investing in sustainable production methods like vertical farming and mycelial fermentation, and developing comprehensive safety databases for long-term consumption. The convergence of these factors positions mushroom beverages not as a niche health product, but as a major category in the global functional food market, projected to capture 15% of the $280 billion industry within the next decade. As research continues to uncover novel applications—from mental health support to healthy aging—these products have the potential to redefine our understanding of food as medicine while creating new opportunities across the value chain, from sustainable agriculture to precision nutrition. The roadmap forward is clear: through rigorous science, thoughtful innovation, and responsible marketing, mushroom beverages can achieve their potential as both a commercial success and a force for improved public health.
